# Exploration of Type III effector *Xanthomonas* outer protein Q (XopQ) inhibitor from *Picrasma quassioides* as an antibacterial agent using chemoinformatics analysis

**DOI:** 10.1371/journal.pone.0302105

**Published:** 2024-06-18

**Authors:** Prasanna D. Revanasiddappa, H. G. Gowtham, Chikkanna G. S., Suchithra Gangadhar, Satish A., M. Murali, Chandan Shivamallu, Raghu Ram Achar, Ekaterina Silina, Victor Stupin, Natalia Manturova, Ali A. Shati, Mohammad Y. Alfaifi, Serag Eldin I. Elbehairi, Shiva Prasad Kollur, Kestur Nagaraj Amruthesh

**Affiliations:** 1 Department of Biotechnology, Siddaganga Institute of Technology, Tumkur, India; 2 Department of Studies and Research in Food Science and Nutrition, KSOU, Mysuru, Karnataka, India; 3 Department of Home Science, ICAR Krishi Vigyan Kendra, Kolar, India; 4 Department of Clinical Nutrition and Dietetics, Sri Devaraj Urs Academy of Higher Education and Research, Kolar, Karnataka, India; 5 Department of Studies in Botany, University of Mysore, Mysuru, Karnataka, India; 6 Department of Biotechnology and Bioinformatics, School of Life Sciences, JSS Academy of Higher Education & Research, Mysuru, Karnataka, India; 7 Division of Biochemistry, School of Life Sciences, JSS Academy of Higher Education and Research, Mysuru, Karnataka, India; 8 Department of Human Pathology, I.M. Sechenov First Moscow State Medical University (Sechenov University), Moscow, Russia; 9 Department of Hospital Surgery, NI. Pirogov Russian National Research Medical University, Moscow, Russia; 10 Biology Department, Faculty of Science, King Khalid University, Abha, Saudi Arabia; 11 Tissue Culture and Cancer Biology Research Laborotory, King Khalid University, Abha, Saudi Arabia; 12 School of Physical Sciences, Amrita Vishwa Vidyapeetham, Mysuru Campus, Mysuru, Karnataka, India; Ahram Canadian University, EGYPT

## Abstract

The present study was focused on exploring the efficient inhibitors of closed state (form) of type III effector *Xanthomonas* outer protein Q (XopQ) (PDB: 4P5F) from the 44 phytochemicals of *Picrasma quassioides* using cutting-edge computational analysis. Among them, Kumudine B showed excellent binding energy (−11.0 kcal/mol), followed by Picrasamide A, Quassidine I and Quassidine J with the targeted closed state of XopQ protein compared to the reference standard drug (Streptomycin). The molecular dynamics (MD) simulations performed at 300 ns validated the stability of top lead ligands (Kumudine B, Picrasamide A, and Quassidine I)-bound XopQ protein complex with slightly lower fluctuation than Streptomycin. The MM-PBSA calculation confirmed the strong interactions of top lead ligands (Kumudine B and QuassidineI) with XopQ protein, as they offered the least binding energy. The results of absorption, distribution, metabolism, excretion, and toxicity (ADMET) analysis confirmed that Quassidine I, Kumudine B and Picrasamide A were found to qualify most of the drug-likeness rules with excellent bioavailability scores compared to Streptomycin. Results of the computational studies suggested that Kumudine B, Picrasamide A, and Quassidine I could be considered potential compounds to design novel antibacterial drugs against *X*. *oryzae* infection. Further *in vitro* and *in vivo* antibacterial activities of Kumudine B, Picrasamide A, and Quassidine I are required to confirm their therapeutic potentiality in controlling the *X*. *oryzae* infection.

## Introduction

*Xanthomonas oryzae* is a bacterial pathogen that causes rice’s most destructive leaf blight disease worldwide [1]. It produces a wide range of virulence factors in the host cells, including type III secretion dependent effectors, inducible extracellular enzymes, exopolysaccharides and iron chelating siderophores, which are all necessary for its virulence. The evidence suggests that this pathogen regulates the production of virulence factors through the quorum sensing mechanism of the diffusible signal factor system [2]. The type III secretion system of *X*. *oryzae* directly delivers effector proteins into host cells, which are its virulence factors that promote bacterial pathogenesis by disrupting various cellular processes [3]. The type III effector *Xanthomonas* outer protein Q (XopQ) exhibited nucleoside hydrolase (hydrolytic) activity. The XopQ is a secreted effector protein that plays a crucial part in the pathogen’s ability to deceive the rice plants’ immune system because it disrupts or modifies the plant’s signalling pathways through cell wall damage [4]. However, the XopQ of *X*. *oryzae* suppresses the plant defense responses due to the interaction of bacterial type-III effectors with the cognate 14-3-3 proteins (*viz*., Gf14f and Gf14g) of rice during the infection process [5]. The XopQ interferes with the suppression of both effector-triggered immunity (ETI) and PAMP-triggered immunity (PTI) to promote the virulence of *X*. *oryzae* on rice [6]. Determining the crystal structure of XopQ protein from *X*. *oryzae* in a complex with adenosine diphosphate ribose (ADPR) is a major achievement in understanding the molecular mechanisms responsible for the functional role of protein [3]. This complex structure provides valuable insights into how XopQ protein interacts with ADPR and its effects on regulating Ca^2+^-mediated immune response in the host plant. Therefore, inhibiting XopQ protein may be one of the strategies to control the growth of *X*. *oryzae* thereby inducing rice defense responses even during the infection.

The use of biological control agents, antibiotics and cultural practices are some of the traditional management strategies employed against the disease that are still unsuccessful, especially when the disease is in an epidemic state [7]. Therefore, the effective management of plant bacterial diseases is the most pressing global challenge nowadays due to the lack of efficient chemicals and bactericides, high pathogen versatility, significant racial differences and the development of antibiotic resistance [8]. Hence, there is a demand for an alternate, environmentally sustainable method of controlling phytopathogen by utilizing naturally occurring phytochemicals. The bioactive phytochemicals are the secondary metabolites produced naturally by plants to protect them from harmful microbial pathogens [9]. Numerous phytochemicals (such as flavonoids, alkaloids, saponins, tannins, terpenoids, steroids, quinones, glycosides, etc.) have been isolated from the thousands of medicinal plants that have traditionally been exploited for managing many diseases [10, 11]. They may be more effective in treating various human diseases and have no or fewer toxic side effects than contemporary drugs. Therefore, the best pharmacological potential for treating diseases may be the phytochemicals because they have been consumed in large quantities in the daily diet since ancient times and still have antimicrobial, antioxidant, anti-inflammatory, and anti-carcinogenic effects at low doses.

The small shrub *Picrasma quassioides*, also known as nigaki bitterwood, is grown commonly in the Indian Himalayan regions of Asia [12]. It has been particularly used as an Asian traditional medicine to treat different diseases, including malaria, fever, gastritis and pediculosis, etc. It can strengthen the stomach, remove intestinal parasites, eliminate dampness, and promote pus discharge [13]. The major phytochemicals extracted from *P*. *quassioides* are associated with the flavonoids, alkaloids (*viz*., cathinone, β-carbolines and cinnamamides), neolignans and triterpenoids (*viz*., apotirucallanes, quassinoids, and tirucallanes), which intensively have many potential pharmacological activities such as antioxidant, anticancer, antimicrobial, anti-diarrheal, anti-inflammatory, neuroprotective, anti-amoebal, antihypertensive, anti-asthma, anti-osteoporosis and anti-parasitic activities [14, 15]. For instance, the active chemicals from the stem of *P*. *quassioides* (i.e., a well-known traditional Chinese medicine known as Kumu) were isolated and identified [16]. These chemicals belonged to β-carbolines or their dimers and exhibited strong antimicrobial activities against *Escherichia coli* and *Staphylococcus aureus*. This result supports the medicinal application of the extract of Kumu (*P*. *quassioides*’ stem) as an antibacterial agent and it offers an intriguing foundation for advancing the development of powerful and targeted antibacterial agents. The literature suggests that the study on *P*. *quassioides* is associated with a larger scientific effort to identify bioactive compounds with distinctive structural properties from natural sources [17, 18].

Chemoinformatics utilizes structurally diverse phytochemicals, eventually expediting the drug development pipeline [19, 20]. The computational/ *in silico* methods are crucial in understanding the proper inhibitory potential against targeted protein receptors by studying the protein-ligand interaction between the ligands and target proteins/enzymes [21–23]. The powerful *in silico* high-throughput screening tool is widely employed to quickly screen the maximum number of compounds that identify the effective drugs and minimize the number of compounds for *in vitro* and *in vivo* screening. This study was intended to identify the inhibitory phytochemicals from *P*. *quassioides* against the XopQ protein of *X*. *oryzae* as the promising antibacterial drug candidates using *in silico* methods. The specific objectives of the study were the virtual screening of 44 phytochemicals extracted from *P*. *quassioides* against XopQ protein by molecular docking studies, evaluating the protein-ligand complex stability through molecular dynamics (MD) simulations and assessing the structure-activity relationship (SAR) and absorption, distribution, metabolism, excretion, and toxicity (ADMET) analysis of the potential lead compounds.

## Materials and methods

### Preparation of ligands

The 44 phytochemicals extracted from *P*. *quassioides* [14] served as ligands for virtual screening against the target receptor (XopQ protein). The antibiotic Streptomycin was used as a viability control [24] while adenosine diphosphate ribose (ADPR), which is a native ligand that has been complexed with XopQ protein (PDB: 4P5F) was used as a positive control for comparing the results. The two-dimensional (2D) and three dimensional (3D) structures of chemicals were acquired from the PubChem database in the form of structured data format (SDF) files [25]. The MarvinSketch software (version 18.3.0) was used to draw the unknown chemical structures in the database. The SDF files were converted into the Protein Data Bank (PDB) file format with OpenBabel software (version 2.3.2). The PRODRG online server was utilized to optimize the geometries of ligand PDB files before molecular docking.

### Preparation of target protein structure

The crystal structure of type III effector protein XopQ from *X*. *oryzae* complexed with ADPR (PDB: 4P5F at 2.10 Å resolution) was acquired from the Research Collaboratory for Structural Bioinformatics (RCSB) PDB database in PDB format and considered as a target protein receptor [3]. This crystal structure is a protein dimer composed of open and closed conformations. The structural superposition of the closed state (form) of XopQ protein, which contains calcium (Ca^2+^) ion and ADPR at its active binding site, was used for the molecular docking experiment. First, the protein structure was processed by deleting unnecessary water molecules from the protein and adding hydrogen atoms, missing residues and appropriate charges to the protein using the MODELLER10.4 program (https://salilab.org/modeller/10.4/release.html). Finally, the protein structure was subjected to energy minimization with Swiss-PDB Viewer (version 4.1.0) and further validated before protein-ligand docking.

### Molecular docking analysis

CASTp 3.0 online server was used to determine the protein’s active site [26]. Molecular docking was executed with AutoDock Vina 1.2.0 implicated in PyRx 0.8 virtual screening software, which simultaneously performs the docking of multiple ligands [27]. The cubical grid dimensions were fixed to 51 × 57 × 81 Å xyz directions with a grid spacing of 0.375 Å around the binding sites of XopQ protein. The docking was performed with the default exhaustiveness of 100. The docking scores were obtained regarding the binding energy, usually expressed in kilocalories per mole (kcal/mol). The conformation of compounds with the least binding energy was considered the best-docked pose and only used for subsequent analyses. The protein-ligand interaction in the best-docked complex was analyzed using BIOVIA Discovery Studio Visualizer 4.5. The accuracy of the docking methodology was further validated by performing the re-docking with the best compounds and XopQ protein.

### Docking validation

To quantify the results obtained through molecular docking, 2D and 3D interaction plots were generated depicting the interacting zone of ligands with proteins at the active site. These interaction plots were generated by Ligplus [28], a program that gives 2D and 3D interaction plots for the submitted PDB file.

### Binding pocket prediction

The protein binding pocket was analyzed using the Fpocket platform (https://fpocket.sourceforge.net/) to predict the ligand-binding pocket [29]. The predicted active site of protein was supported with the CASTp 3.0 server.

### Molecular dynamics simulation

The top lead ligands (Kumudine B, Picrasamide A, and Quassidine I)-bound and standard antibiotic (Streptomycin)-bound, as well as the native ligand (ADPR)-bound XopQ (represented as APO complex) protein, were subjected to molecular dynamics (MD) simulations. The MD simulations were accomplished for the docked protein-ligand complexes with the GROMACS 2020.4 simulation package using the CHARMM36 force field to verify their structural stability and binding mode in a dynamic system [30]. The CHARMM general force field (CGenFF) program was initially used to generate the protein topology [31]. The GROMACS pdb2gmx module was used to convert the.pdb file into a.gro file. Subsequently, the solvation of docked complexes was achieved with the SPC/E water model, followed by neutralization with the addition of a counter number of Na^+^ and Cl^−^ions. The steepest descent algorithm (5000 steps) was exploited for energy minimization of the neutralized solvated system. Furthermore, the system was equilibrated for a 100 ps time scale in the separate NVT ensemble, followed by the NPT ensemble according to the LINCS algorithm. Finally, the MD simulations were executed at 300 ns under the simulating system’s constant pressure (1 bar) and temperature (300 K). After performing the MD simulations, the LINCS algorithm generated the final conformational transitions in MD trajectories. The generated simulation trajectories, including root mean square deviation (RMSD), root mean square fluctuation (RMSF), radius of gyration (Rg), solvent-accessible surface area (SASA), and the number of hydrogen bonds formed, were examined for comprehending the behavior of complex in the water environment. The plots obtained from these trajectories were visualized in the xmgrace 5.0.5 software.

### Binding free energy calculation

The Molecular Mechanics Poisson Boltzmann Surface Area (MM-PBSA) method was employed to calculate the binding free energy from the MD trajectories obtained [32]. The g_mmpbsa program was utilized to perform MM-PBSA calculation in GROMACS 2020.4, which gives the results including electrostatic energy, van der Waal’s energy, and polar solvation energy. The binding free energy of protein-ligand interaction in the solvent system was calculated by using the following formula:

$$\Delta {\text{G}}_{\text{Binding}}={\text{G}}_{\text{Complex}}–\left({\text{G}}_{\text{Protein}}+{\text{G}}_{\text{Ligand}}\right)$$

where G represents the Gibbs free energy, G_Complex_ represents the total free energy of the protein-ligand complex and G_Protein_ and G_Ligand_ represent the total free energies of protein and ligand, respectively, in the solvent.

### Principal component analysis (PCA)

The Bio3D trajectory analysis package achieved the PCA to identify the dominant motions and interactions of protein-ligand complexes [33]. Each complex’s motion type was observed and plotted as a PCA plot on the R platform.

### Structure-activity relationship (SAR) analysis

The SAR was analyzed using the DataWarrior software (version 5.5.0) with a default FragFp descriptor that calculates the chemical structure similarity value between the compounds to filter the large collection of biologically active compounds [34]. The structural similarity chart showing the structure similarity of phytochemicals used in the present study was based on their binding behaviour and canonical SMILES structure (S1 Table).

### Absorption, distribution, metabolism, excretion, and toxicity (ADMET) properties

Physicochemical descriptors and ADMET properties of the most promising compounds were calculated using the pkCSM signatures [35]. The physicochemical parameters predicted are the molecular weight, partition coefficient (Log P), number of rotatable bonds, number of hydrogen bond acceptors and donors, and topological polar surface area (TPSA). The BOILED-Egg predictive model was used to estimate the human intestinal absorption and brain penetration of compounds simultaneously through the computation of lipophilicity (WLOGP) and polarity (TPSA) in the SwissADME web server [36]. The SwissADME server was also exploited to calculate the bioavailability score of selected compounds based on the enzyme inhibition score [37]. It was recommended that the drug be more active if the bioavailability score is ≥ 0, while it is moderately active if the scores are between −0.5 and 0, and it is to be considered inactive if the score is < −0.5.

## Results and discussion

Since secondary metabolite production frequently produces substantial biosynthetic pathway intermediates, finding ample structurally related compounds in the plant extracts is instructive. The rationale of the present study was to explore the pool of 44 phytochemicals of *P*. *quassioides* as efficient inhibitors of the closed state of XopQ (PDB: 4P5F) protein by employing cutting-edge computational analysis. The study will also reduce the time phase, but they introduce highly efficient drugs with efficient biological activity and minimal side effects towards specific pathogens.

### Molecular docking

**Fig 1:**
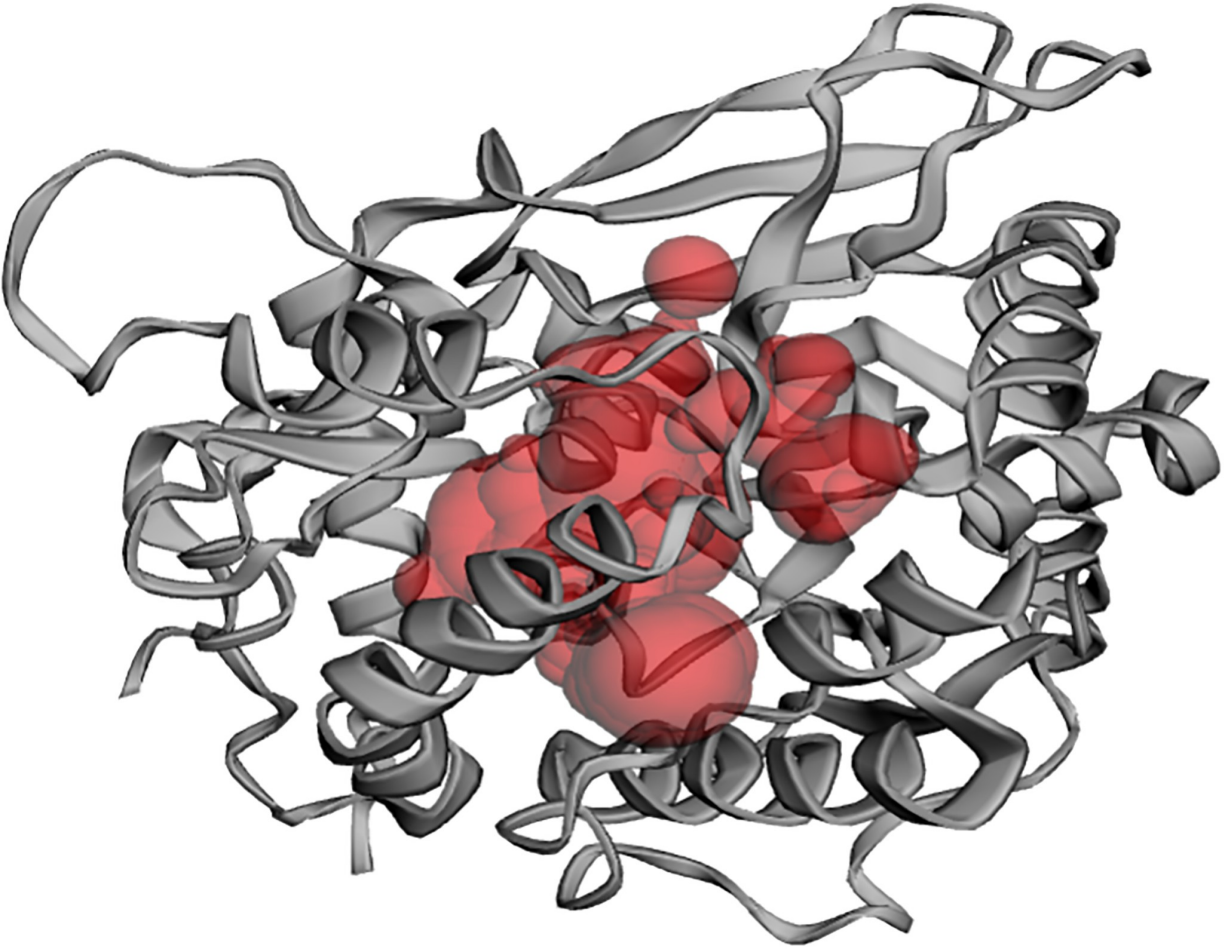
Active site of the closed state of XopQ protein (PDB: 4P5F) predicted from the CASTp 3.0 web server. The active site region of the protein is shown as red; transparent space fills the form, while the protein is shown as a cartoon representation (Color: Dark grey).

Molecular docking is a well-established and reliable *in silico* structure-based method widely utilized in the structural biology field of computer-aided drug design [38–40]. It facilitates the discovery of new therapeutic compounds by predicting protein-ligand interactions at the molecular level without prior knowledge of the chemical structures of other selective receptor modulators [41]. It is well known that the functional activity of enzymes will be terminated if the small ligand molecules block their active binding sites. Fig 1 demonstrates the active site of the closed state of the XopQ protein identified using the CASTp server. The possible binding pocket residues identified using the CASTp server is shown in Table 1.

**Table 1:** Residues identified in the enzyme’s binding pocket as predicted by all three prediction tools.

CASTp Residues	Fpocket Residues	Ligplus Residues	Residues in common
ASP35, LYS38, ASP39, PRO40, ASP41, ASP42, LEU67, GLN106, ARG108, GLU109, HIS110, LYS112, ASP182, ALA184, ARG196, ALA197, TYR198, ASN200, HIS205, ARG208, TYR230, LYS231, THR235, PRO236, ALA237, GLY241, ALA243, ASN271, LEU272, PHE282, ARG283, THR284, ARG283, THR284, GLN289, GLN291, PRO293, GLN298, LEU302, PHE304, ALA306, TRP308, THR312, ASN315, LEU316, TYR317, ASP318, GLU359, ARG362, LYS370	ASP35, ASP39, MET178, ASP182, ARG196, TYR198, ASN199, LYS226, TYR230, LYS231, GLU240, LEU264, TRP280, PHE282, ARG283, THR284, GLN298, TRP308, VAL311, ASN315, ASP318, GLU359, ARG362, LYS370	GLU227, TYR230, LYS231, ALA233, ARG283, THR235, THR284, ALA287, ALA288, GLN289, PRO290, GLN291, ALA295, ALA296, ASP297, GLN298, THR312, LYS313, HIS352, GLU359, ARG362	TYR230, LYS231, ARG283, THR284, GLN298, GLU359, ARG362

**Fig 2:**
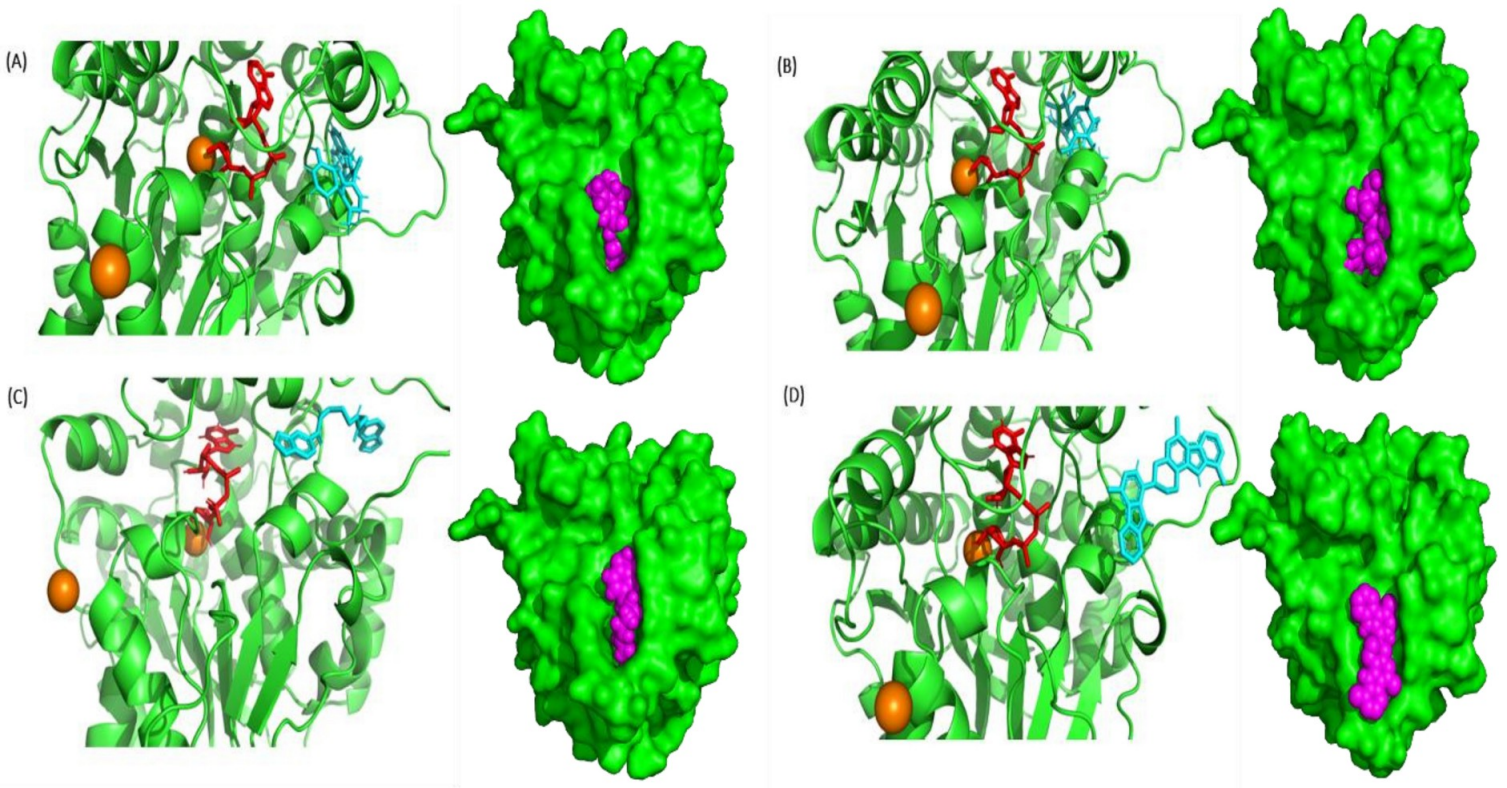
Binding of best ligands with XopQ protein. The binding of (A) Kumudine B (cyan) with ADPR (red) and Ca^2+^ (orange) in the binding pocket of XopQ protein (shown as a green color cartoon); (B) Binding of Streptomycin (cyan) with ADPR (red) and Ca^2+^ (orange) in the binding pocket of XopQ protein (shown as a green cartoon). With a similar color code; (C) Picrasamide A complex and (D) Quassidine I were shown in the protein’s binding pocket. The respective protein-ligand conformation is shown in green surface with ligands in magenta sphere against their respective binding pockets.

A total of 44 phytochemicals of *P*. *quassioides* were docked against the closed state of XopQ protein (PDB: 4P5F) using cutting-edge chemoinformatics techniques like molecular docking. The XopQ is a homodimer with a sequence length of 370 residues in each protein chain and a native ADPR ligand with two calcium ions. The docking scores obtained from the phytochemicals of *P*. *quassioides* docked with the closed state of XopQ protein were portrayed in Table 2. Surprisingly, Kumudine B was the most potent compound against the closed state of XopQ protein, with a good docking score of −11.0 kcal/mol compared with the standard drug (Streptomycin). Competent inhibitors typically contain adequate hydrogen bonds, electrostatic interactions, and hydrophobic interactions, which contribute to their strong affinity for the active site of targeted proteins during protein-ligand interactions [42]. New hydrogen bonds can be formed between the proteins and ligands during protein-ligand interaction by breaking hydrogen bonds with water molecules. Kumudine B showed the conventional hydrogen bond interaction with THR284, GLN289, and GLN298 amino acids at the active bonding sites of the XopQ protein. The binding interactions of the best-docked compounds (Kumudine B, Picrasamide A and Quassidine I) and standard drug (Streptomycin) with the XopQ protein are illustrated in Fig 2.

**Table 2:** Docking scores of phytochemicals from *P*. *quassioides* and standard drug against the closed state of XopQ protein (PDB: 4P5F) of *X*. *oryzae*.

Sl. No.	Compound Name	PubChem CID	Docking score (kcal/mol)
1.	Bruceantin	CID_5281304	– 8.7
2.	* Kumulactone A	-	– 9.2
3.	* Kumulactone B	-	– 7.8
4.	* Picrasinoside J	-	– 9.4
5.	* Picrasinoside K	-	– 9.4
6.	Quassine	CID_65571	– 7.7
7.	Neoquassine	CID_72964	– 7.8
8.	Simalikalactone D	CID_6711208	– 7.7
9.	Simalikalactone E	CID_53324651	– 7.3
10.	Picrasin A	CID_185611	– 8.8
11.	Picrasin B	CID_12313355	– 8.6
12.	Picrasin C	CID_182145	– 7.1
13.	* 12-hydroxyquassin	-	– 8.9
14.	Nigakihemiacetal A	CID_441803	– 7.6
15.	Nigakilactone A	CID_10452259	– 7.8
16.	Nigakilactone B	CID_12313347	– 7.4
17.	* 1-hydroxymethyl-8-hydroxy-beta-carboline	-	– 6.8
18.	Dehydrocrenatidine	CID_5318875	– 6.6
19.	* Quassidine I	-	– 9.5
20.	* Quassidine J	-	– 9.5
21.	Picrasidine I	CID_5324360	– 6.7
22.	* 6,12-dimethoxy-3-ethyl-beta-carboline	-	– 6.5
23.	* Kumudine A	-	– 8.9
24.	* Kumudine B	-	– 11.0
25.	Nigakinone	CID_5320161	– 7.6
26.	Methyl nigakinone	CID_638215	– 7.3
27.	Picrasidine O	CID_5320558	– 8.0
28.	* 4,5-dimethoxy-10-hydroxycanthin-6-one	-	– 8.3
29.	* 8-hydroxycanthin-6-one	-	– 7.5
30.	* Picrasmalignan A	-	– 9.4
31.	Dehydrodiconiferyl alcohol	CID_5372367	– 7.0
32.	Picraquassioside C	CID_10077272	– 7.6
33.	* Picraquassin A	-	– 9.2
34.	* Picraquassin B	-	– 7.8
35.	* Picraquassin C	-	– 8.6
36.	* Picraquassin D	-	– 8.4
37.	* Picraquassin E	-	– 9.2
38.	* Picraquassin I	-	– 9.1
39.	* Picraquasssin J	-	– 9.0
40.	* Picraquassin K	-	– 8.7
41.	* Kumuquassin A	-	– 9.2
42.	* Kumuquassin B	-	– 9.0
43.	* Kumuquassin C	-	– 9.1
44.	* Picrasamide A	-	– 9.8
45.	Streptomycin [Standard drug]	CID_19649	– 8.4

* Structures of phytochemicals were not found in any of the databases and were drawn using MarvinSketch software, according to Mohd Jamil et al. [14] and used in the present study.

In virtual screening, the most potent compounds (Kumudine B, Picrasamide A and Quassidine I) and standard drug (Streptomycin) were then accelerated to perform the molecular re-docking with the XopQ protein. The two more compounds, Picrasamide A and Quassidine I, with a better binding score, as shown in Table 1, were also considered for mutual comparison against Kumudine B. The virtually screened 2500 rifampicin analogs from Zinc Database were docked into the active site of regulation of pathogenicity factor F (RpfF) protein in *X*. *oryzae* with AutoDock Vina in PyRx virtual screening tool and the docking analysis described that the compounds ZINC03056414, ZINC03205310, ZINC08673779, ZINC09100848, ZINC09729566, ZINC11415953, ZINC12810788, ZINC24989313, ZINC27441787 and ZINC32739565 were found to have best binding energies than reference compound [43]. The virtual screening of the library of 318 phytochemicals with both rigid and flexible molecular docking against targeted peptide deformylase of *X*. *oryzae* showed that 14 compounds derived from different plants have excellent binding energy when compared to reference molecule (−7.7 kcal/mol) [44].

The binding pocket is where the protein’s active site, which holds the small molecules in it, is assumed to be present. The binding pocket of the closed-form XopQ protein was determined through the CASTp web server. This web server determines the pocket of the submitted protein structure by running a probe of 1.4 Å radius. The probe rolls over the submitted query structure and determines the key catalytic residues. The pocket area determined by CASTp of XopQ protein was measured to be 540.656 Å^2^, comprising 48 amino acids. These residues are identified as the binding pocket residues suitable for holding the ligand. Out of 48 amino acids, few residues may act as catalytic residues, which play an important role in the protein activity. In addition, we also performed binding pocket analysis using the Fpocket tool, which identifies the binding pocket of small molecules in the protein over molecular dynamics trajectory data. Fpocket is an open-source platform based on Voronoi tessellation and relies on the concept of alpha spheres. Fpocket’s algorithm mainly consists of three steps, which start by identifying the alpha spheres ensemble from the submitted protein structure, then it identifies the closest cluster of spheres (identifying the binding pocket), and finally completes its job by calculating the properties of atoms in the binding pocket.

**Fig 3:**
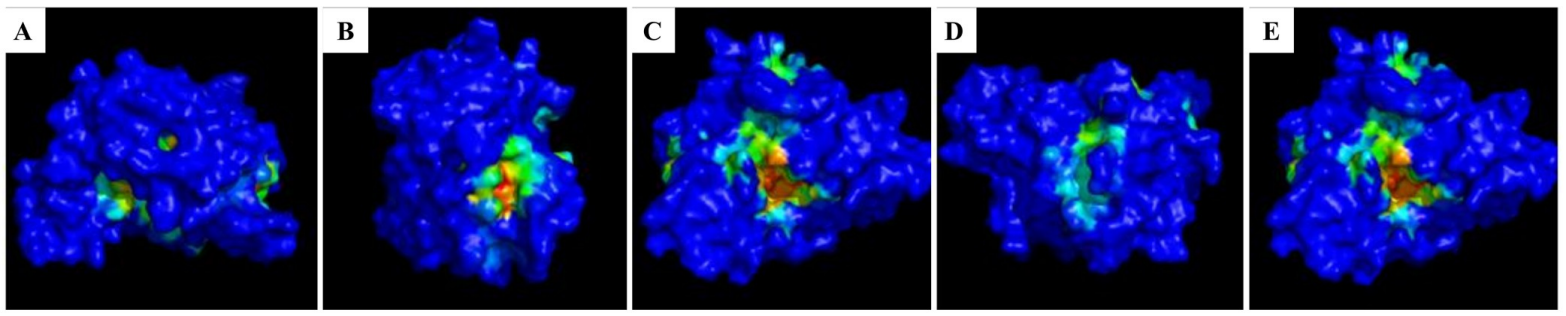
Binding pocket detected by Fpocket. A: Apo complex; B: Streptomycin complex; C: Kumudine B complex; D: Picrasamide A complex; E: Quassidine I complex where the blue colored surface represents the entire protein with orange and green color patches indicating the binding pocket of the ligands. The binding pocket was observed to be similar in most of the complexes.

The MD run data were provided as the input for the Fpocket tool to predict the binding pocket. The result showed that the binding pocket detected by the Fpocket tool was almost similar in place to the pocket identified by the CASTp server. However, the area identified as the binding pocket region for all complexes averaged around 250 Å^2^ (Fig 3). Despite the reduction in the area of the binding pocket, around 15 residues were found to be common among both tools. Here, we presume that the catalytically important residues for the protein’s function will be inclusively present in the residues identified by pocket-finding tools. For example, the residues, namely THR284, GLN289, and GLN298 identified by the CASTp and Fpocket server, were observed to present in the docked complex and found to interact with the ligand. Further, this presumption is validated by analyzing 2D and 3D interaction plots generated using Ligplus on the docked complexes generated from docking results.

### Catalytic residues

**Fig 4:**
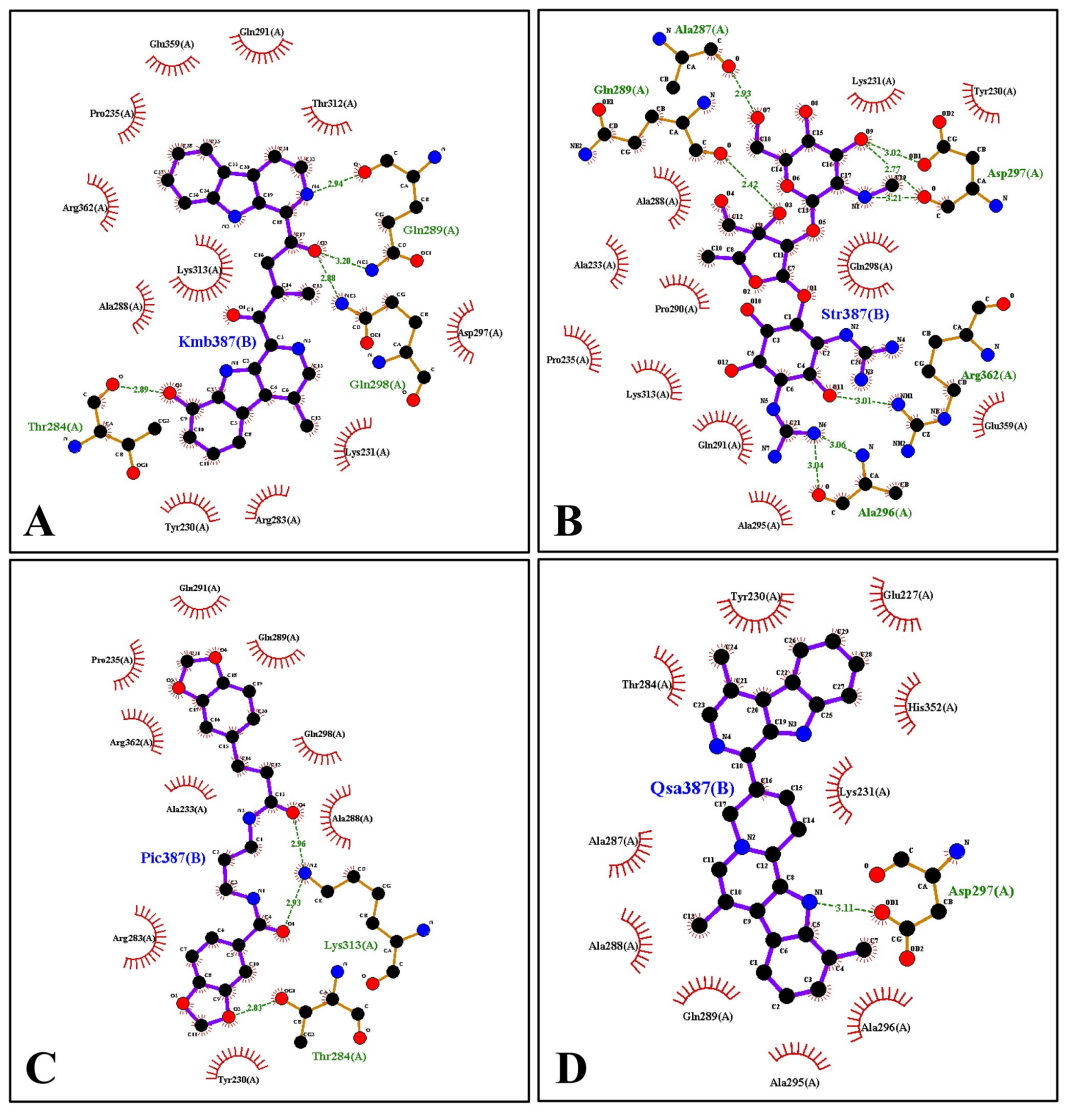
2D interaction plot of Kumudine B (A); Streptomycin (B); Picrasamide A (C) and Quassidine I (D) in the XopQ docked complex indicating the residues interacting with the respective ligands. Each ligand is shown in a stick representation with interacting residues in half-sun-shaped curves.

**Fig 5:**
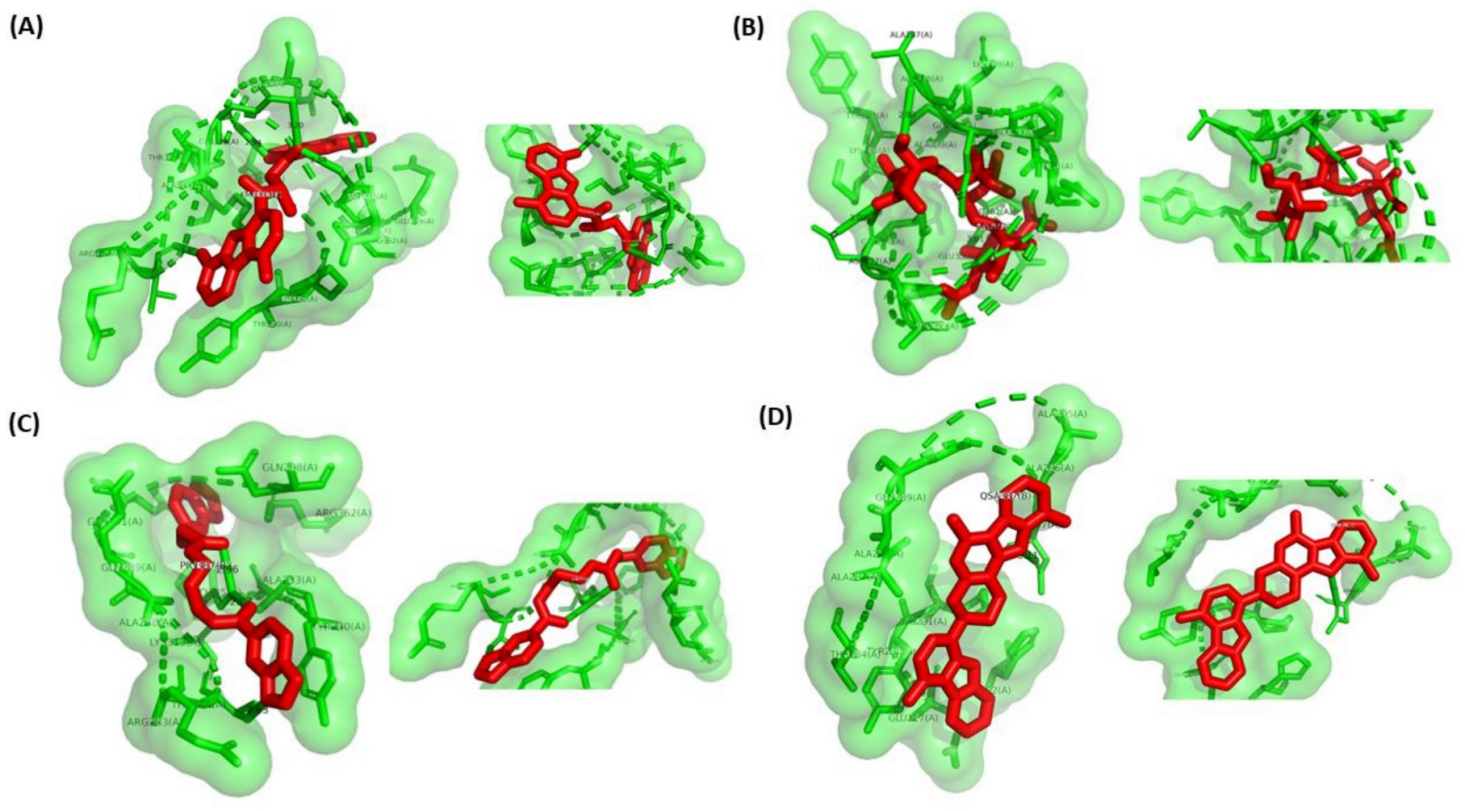
3D interaction plot of Kumudine B (A); Streptomycin (B); Picrasamide A (C) and Quassidine I (D) in the XopQ docked complex indicating the residues interacting with the respective ligands. Each ligand is shown in red stick representation with interacting residues as green sticks with a transparent space-fill format.

To further validate the presence of catalytic residues at the active site in the docked complex, the 2D and 3D interaction plots were generated on the PDB structure obtained upon docking. The four docked complexes, namely protein possessing Quassidine I, Kumudine B, Picrasamide A, and Streptomycin, were subjected to Ligplus to generate 2D and 3D interaction plots. Ligplus initially unrolls the 3D structure of the ligand around rotatable bonds into 2D. Then, the atoms connected to the rotatable bonds come to the same plane, representing the ligand interaction. The ligands’ 2D and 3D interaction plots are shown in Figs 4 and 5. The Figures show that all four compounds have active site residues predicted around them (Table 1). However, it is also observed that there are a few instances where the residues interacting with the ligand during docking may not be present in the results of the CASTp tool. It can be directly correlated to the conformational sampling of the ligand during docking in the active site of the protein, which can sample the residues that are not identified during the active site pocket prediction tool. Here, the 2D interaction Ligplus plot of Kumudine B has identified 14 residues in the interacting zone of the ligand with the protein. Of these residues, LYS313, ALA288, and LYS231 were not identified by the CASTp server but are present in the interacting zone of the docked complex. Majorly, 11 residues out of the 14 residues identified by Ligplus were identified by CASTp, confirming the reliability of the docked complex. Also, the previous literature [3] indicated residues like Tyr279, Leu342, Leu345, Trp361, and Phe366 were found to interact with ADPR moiety in the enzyme. As ADPR moiety is very near the ligand binding domain, the active site residues are assumed to be present around the residues. Similarly, for Streptomycin, Picrasamide A, and Quassidine I, 8 residues, 11 residues, and 4 residues, respectively, were found commonly in both Ligplus and CASTp results. Thus, it confirms the reliability of the docked complex at the active site region of the protein and validates the presence of catalytic residues.

### MD simulations

**Fig 6:**
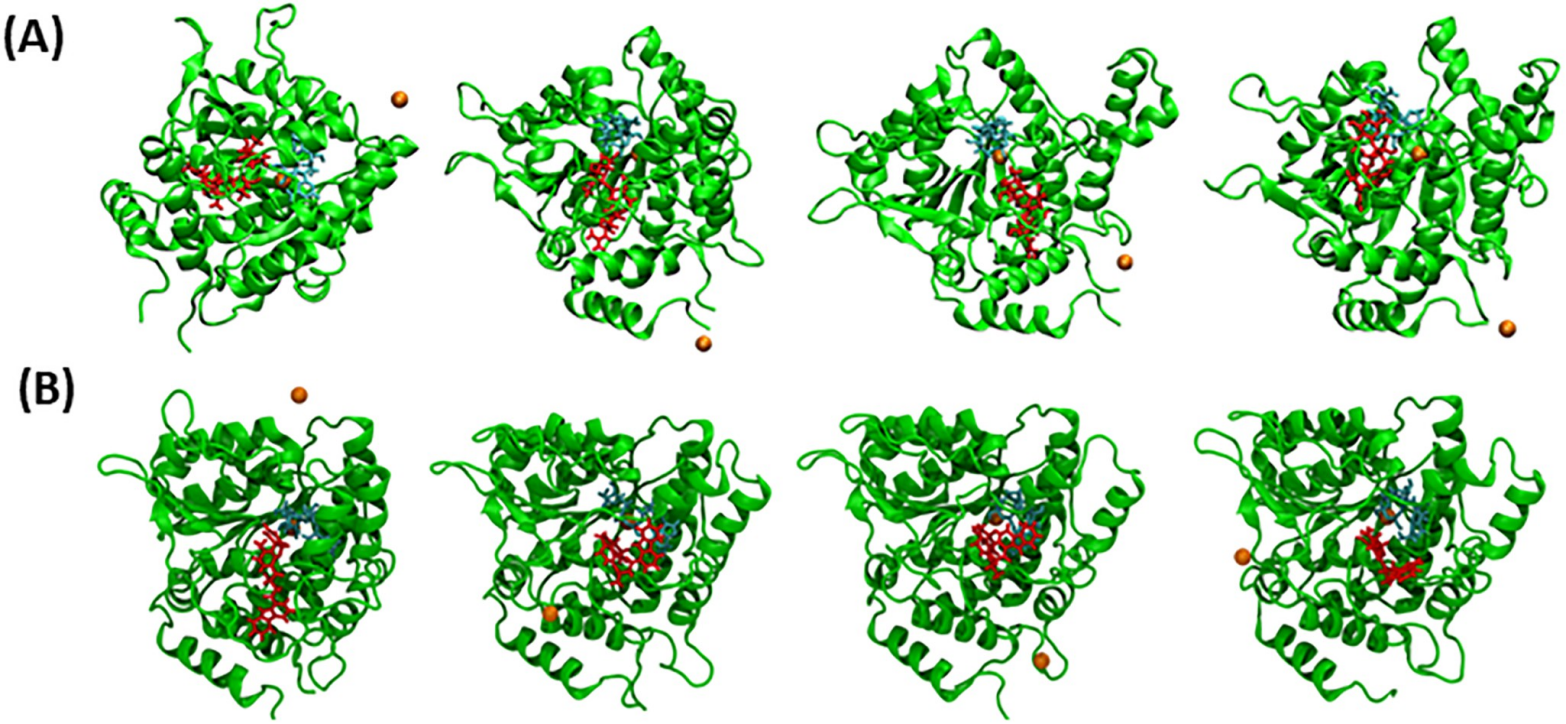
Snapshot of (A) Streptomycin and (B) Kumudine B at 0 ns, 100 ns, 200 ns and 300 ns, respectively. Red: ligand, cyan: ADPR, orange: calcium ions.

The MD simulations are an advanced method that can effectively comprehend the structure-to-function relationship among various biological macromolecule complexes with different small molecules [45, 46]. The MD simulations were run at 300 ns to explore the structural behavior of the protein and further validate the docking scenario of the topmost ligands (Kumudine B, Picrasamide A, and Quassidine I) with the reference drug (Streptomycin) and native ligand (ADPR) complexed with the XopQ protein (APO). The snapshot for the simulated ligand-target complex across specific time intervals (0 ns, 100 ns, 200 ns, and 300 ns) are shown in Fig 6 and S1 Fig to track the conformational and orientation changes.

**Fig 7:**
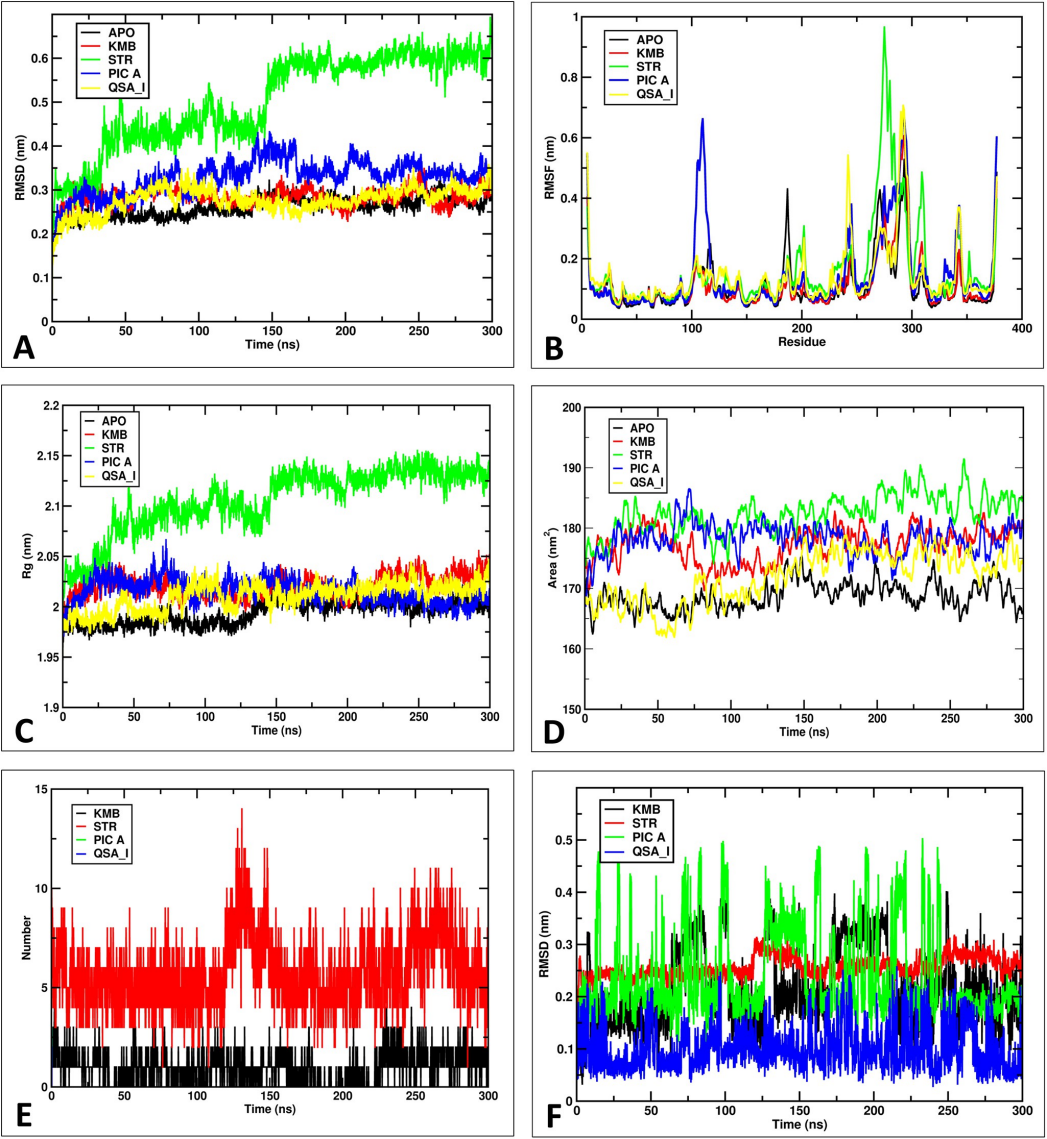
The results of MD simulations experienced over the simulation time. A: RMSD; B: RMSF; C: Radius of gyration; D: SASA; E: H-bond analysis; F: Ligand RMSD.

Next, the RMSD analysis of the protein backbone identified stable regions of ADPR-bound XopQ protein and selected protein-ligand complexes. The RMSD calculation is one of the important factors for analyzing the conformational stability and equilibration of MD trajectories [47]. Deviations of the protein structure from its initial position were calculated and plotted as the time in ns versus RMSD value in nm. The RMSD plot demonstrated that the ADPR-bound XopQ protein complex was stable after 25 ns simulation with an averaged RMSD value of 0.25 nm. Similarly, Kumudine B-XopQ protein complex was also observed to be stable after 25 ns with an average RMSD of 0.27 nm (Fig 7A). Similarly, the RMSD of Picrasamide A, and Quassidine I was found to have an average value of 0.32 nm and 0.27 nm, respectively, typically becoming stable after 75 ns. Meanwhile, Streptomycin-XopQ protein complex was stable from 50–150 ns, then spiked to 0.6 nm and became stable from 160 ns. The results indicate that Kumudine B, Picrasamide A, and Quassidine I attained stability by slightly deviating from the reference structure, indicating a well-equilibrated system. But, in the case of Streptomycin-XopQ protein complex, the RMSD has spiked up to 0.6 nm, indicating the altered stability index of the protein. It is possibly due to the conformational flexibility of Streptomycin sampling wider regions in the binding pocket, making protein-to-sample differential conformational states depicting varied RMSD states.

Subsequently, to understand the protein flexibility over stable RMSD states of the protein, the RMSF analysis was performed to calculate the average fluctuations of protein residues from a reference position [48]. The RMSF plot identified a clear increase in the fluctuation within amino acid regions 250 and 300 for most complexes. Upon careful observation, the region between 250 and 300 was perceived to govern the binding pocket, mostly possessing loops as secondary structural units. Therefore, differential fluctuations were observed for all the ligands depending on the conformational behavior of each ligand. Here, Streptomycin, being a more flexible molecule, was found to influence the binding pocket to fluctuate (the green color RMSF plot with a peak value nearly at 1 nm between the region 250 and 300) to a greater extent when compared to others. Thus, it shows variation in the RMSD plot and highly fluctuating RMSF plot. It was also observed that the RMSF was more in the majority regions of Streptomycin-bound protein, indicating the differential ability of Streptomycin compared to other ligands. Whereas ligands such as Kumudine B, Picrasamide A, and Quassidine I were found to have slightly lower fluctuation compared to Streptomycin, indicating slight restrictions in the conformational sampling of the ligands. It can be directly attributed to the lower number of rotatable bonds in Kumudine B (6 rotatable bonds), Picrasamide A (10 rotatable bonds), and Quassidine I (1 rotatable bond), making them attain a stable state in the binding pocket of the enzyme very quickly compared to Streptomycin which is observed to have a greater number of rotatable bonds (20 rotatable bonds). Thus, reasoning for differential fluctuational states for each ligand (Fig 7B).

The root mean square distance between the atoms of the structure and its center of mass determines the Rg [49]. The Rg value is a key measure to determine the compactness of protein-ligand complexes. The lower Rg values denote more compact protein-ligand complexes and the higher Rg values denote more labile protein-ligand complexes. The Rg was calculated from a point where the center of mass will be concentrated. The average Rg value of ADPR-bound XopQ protein complex was found to be 1.98 nm, Kumudine B-XopQ protein complex was found to be 2.01 nm. The average Rg value of Picrasamide A and Quassidine I XopQ protein complexes were found to be around 2 nm throughout the simulation (Fig 7C). However, the distribution of Rg was observed to vary from the range of 2.1 nm to 2.14 nm in the Streptomycin-XopQ protein complex indicating a less compact molecule in the presence of Streptomycin. This observation can be directly attributed to the ligand’s increased conformational flexibility due to more rotatable bonds. Also, increased RMS fluctuations and increased RMSD are directly correlated with the decreased compactness of the protein, while the remaining three compounds have the optimum compactness, suggesting optimum RMSF and a very stable RMSD.

The SASA of the complexes is an imperative measure for assessing protein stability and folding, where a higher SASA correlates with an increase in protein surface area. In comparison, the lower SASA correlated with a reduction in protein volume [50]. The SASA data were retrieved to study how the protein surface area changed in response to the ligand interaction. The SASA plot showed that the area accessible for solvent during the simulation was observed to be 165 nm^2^, 175 nm^2^, 180 nm^2^,170 nm^2^ and 185 nm^2^ in the ADPR-bound XopQ protein complex, Kumudine B-XopQ protein complex, Picrasamide A-XopQ protein complex, Quassidine I-XopQ protein complex, and Streptomycin-XopQ protein complex, respectively (Fig 7D). As expected, Apo protein was observed to have very low SASA and a very low Rg indicating differential conformational state in the absence of ligands. Here, it was noticed to be more shrunken with very less surface area for solvent accessibility. Further, upon binding with ligands, the protein attained a different Rg and SASA state, indicating the influence of ligands in the binding pocket. Here, Streptomycin bearing more SASA than any other ligands indicates a greater protein conformational flexibility.

Further, to understand the affinity of the complex in the enzyme’s binding pocket, we measured the presence of hydrogen bonds between protein and ligands over simulation time. During the simulation, the hydrogen bonds are formed to ensure the stability and integrity of docked protein-ligand complexes. Hydrogen bonds are the non-covalent interactions between heavy atoms to identify the stability and catalysis of enzymes [42]. Hydrogen bond analysis obtained from the GROMACS module showed that around three hydrogen bonds were formed between Kumudine B and XopQ protein compared to Streptomycin-XopQ protein complex, wherein six hydrogen bonds were observed (Fig 7E). The other two complexes formed a negligible number of hydrogen bonds (only 1 hydrogen bond), hence not visible in the plot. In Streptomycin, the more rotatable hydrogen bonds orient the molecule in a suitable conformation to establish stable interaction by forming more hydrogen bonds with the amino acids in the binding pocket. Meanwhile, the smaller number of rotatable bonds in the remaining molecules induces conformational restrictions, settling in an optimum orientation. Due to a greater number of hydrogen bonds, we presume that Streptomycin’s binding energy in the enzyme’s binding pocket is possibly higher than the binding energies of the other three molecules. However, the optimum properties exhibited by Kumudine B, Picrasamide A, and Quassidine I may serve as an effective alternative to Streptomycin.

Moreover, to substantiate our findings through ligand’s behavior, we performed ligand RMSD. The ligand RMSD was calculated to know the deviations undergone by the ligands that can be correlated to changes in the conformation and orientation of the ligands in the binding pocket (Fig 7F). Here, we found that Streptomycin showed an average deviation of 0.25 nm compared to the average deviation of Kumudine B at 0.28 nm. Kumudine B has shown two trends where the deviation was observed to be 0.3 nm between 50–100 ns and 175–225 ns. In the remaining timeline, the deviation was found to be 0.15 nm. It can be correlated to the orientational behavior of the ligand in the binding pocket, which makes them deviate less at some time and more in the remaining time. However, the overall deviation was in the acceptable range to conclude the conformational flexibility in the binding pocket. The Quassidine I has demonstrated a similar trend to Kumudine B but at a lower scale, marking conformational flexibility. Whereas Picrasamide A has shown greater flexibility possibly due to the presence of more rotatable bonds, making them fluctuate over time.

### Binding free energy calculation

To understand the effect of each ligand in the enzyme’s binding pocket, we examined the relative binding strength for all the molecules using MMPBSA calculations. Table 3 compares each compound’s binding strength in the enzyme’s binding pocket. The MMPBSA calculations are performed on stable RMSD regions of the protein-ligand complexes, covering a minimum of 50 ns simulation time. The results of MMPBSA calculation showed that the ligands Kumudine B, Picrasamide A, Quassidine I, and Streptomycin showed negative binding free energy with the residues in the range of 10 Å distance from the binding pocket. Table 3 shows that the binding energy of Kumudine B was observed to be –27.851 kcal/mol. The Quassidine I also showed a good binding energy of –50.158 kcal/mol. The Picrasamide A exhibited a comparatively acceptable binding energy of –10.923 kcal/mol. Whereas, Streptomycin exhibited a greater binding energy value of –54.230 kcal/mol compared to all other ligands, showing its potential as a standard antibiotic. However, other ligands exhibited a similar potency compared to Streptomycin, suggesting a better alternative obtained naturally as a plant-based product.

**Table 3:** Binding free energy of XopQ protein complexed with lead phytochemicals of *P*. *quassioides* and standard drug.

Category	XopQ protein-Quassidine I complex	XopQ protein-Kumudine B complex	XopQ protein-Picrasamide A complex	XopQ protein-Streptomycin complex
	Values (kcal/mol)			
Van der Waal’s energy	–106.361	–65.420	–4.019	–192.735
Electrostatic energy	–19.647	–19.783	–1.165	–126.389
Polar solvation energy	52.340	67.263	–3.408	289.006
SASA energy	23.510	–9.912	–2.331	–24.113
Binding energy	–50.158	–27.851	–10.923	–54.230

In Kumudine B, Phe304 was observed to engage in displaced Pi-Pi interaction with an average distance of 5 Å. Similarly, Tyr230 participated in edge-to-face Pi-Pi interaction with Kumudine B, maintaining an average distance of 3.15 Å. Additionally, Leu302 and Glu227 interacted with Kumudine B at distances of 5.5 and 3.75 Å, respectively. Likewise, Streptomycin formed stable interactions with Asp297, with an average interacting distance of 2.8 Å, primarily through electrostatic interactions. Lys313 also engaged in slightly weaker electrostatic interaction with Streptomycin, maintaining an average interacting distance of 4.8 Å. In summary, the residues identified by pocket identification tools and MMPBSA analysis clustered around the ligand, forming stable interactions within the van der Waals range of attraction. However, in case of Picrasamide A and Quassidine I, the ligand displayed high flexibility within the pocket, causing the surrounding residues to frequently establish and disrupt interactions. Consequently, while some residues engaged in interactions, others broke them, collectively contributing to the ligand’s stability within the binding pocket.

**Fig 8:**
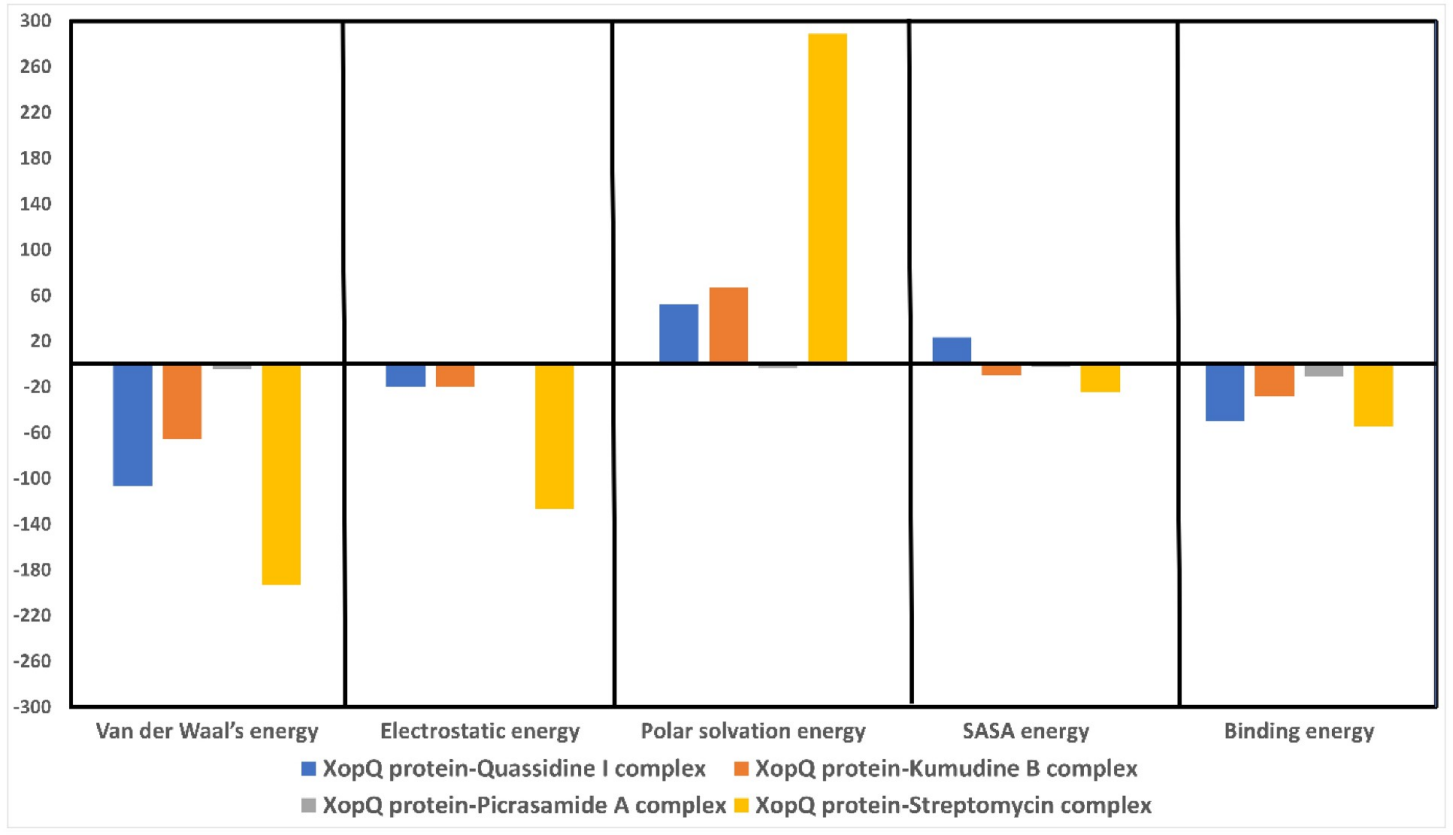
Individual energies contributing to the overall binding energy of XopQ protein complexed with lead phytochemicals.

**Fig 9:**
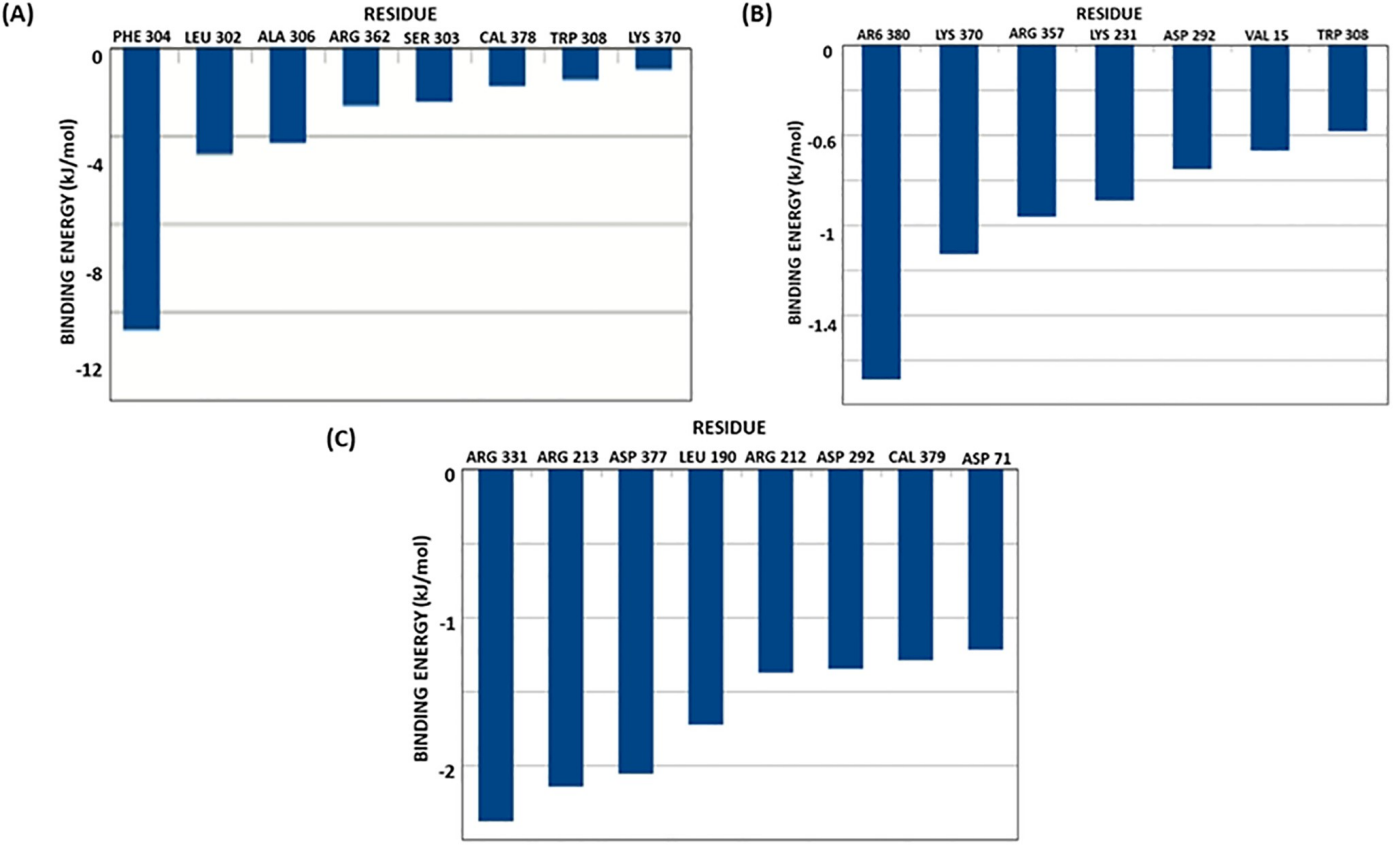
Contribution of individual residues to the overall binding energy of Kumudine B (A); Quassidine I (B) and Picrasamide A (C).

Further, the contribution of individual residues present in the enzyme’s binding pocket toward overall energy is obtained through individual decomposition energy. The decomposition energy plot from Kumudine A, Picrasamide A, and Quassidine I is shown in Fig 8A–8C. From the Figure, it is evident that the binding pocket residues listed by the CASTp server and Fpocket server were mostly present and contributed towards the overall binding energy of the ligands. However, due to a bigger binding pocket (as predicted by the CASTp tool: 540.656 Å^2^), the ligands sampled in their unique conformational states showed very few overlapping residues among them as binding energy contributors. The contribution of individual residues to the overall binding energy of Kumudine B, Picrasamide A and Quassidine I complex is given in Fig 9. Overall, all the proposed natural compounds can act as the proposed lead compounds against Streptomycin specially Kumudine B over Picrasamide A and Quassidine I.

### PCA analysis

**Fig 10:**
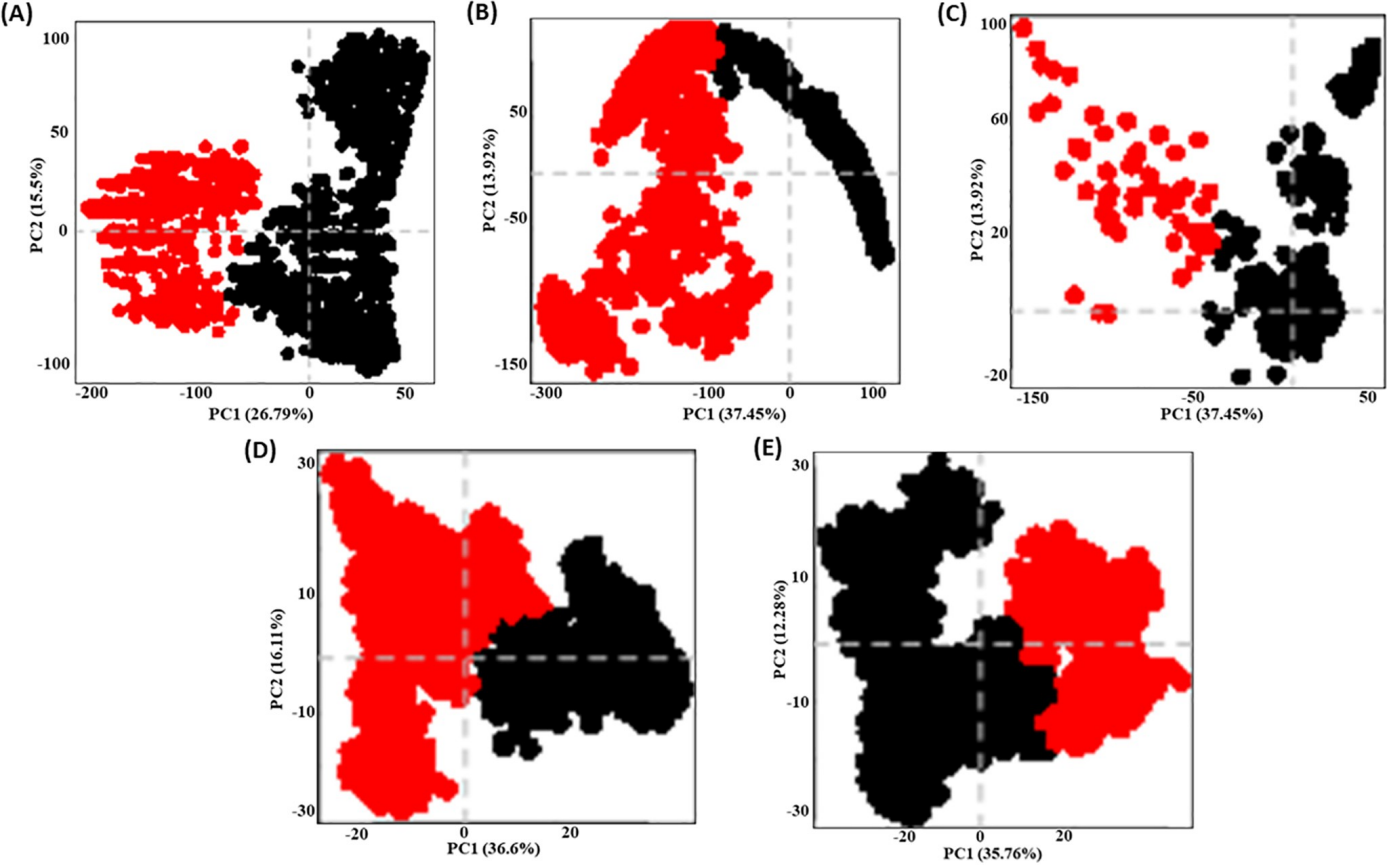
PCA plots of Apo (A); Streptomycin (B); Kumudine B (C); Picrasamide A (D) and Quassidine I (E) complexed with the XopQ protein showing the Principal motion exhibited.

The PCA analysis was done to identify the dominant motions and protein-ligand interactions. The principal components 1 and 2 were calculated using Eigenvectors and diagonal matrix from the GROMACS module, respectively. The PCA plot was then plotted using the Bio3D trajectory analysis package and R studio. The principal component clusters represented in red and black colors are observed to show the dominant motions. PC1 and PC2 are the first and second fluctuations experienced during protein dynamics. The PCA plots show that the Streptomycin-XopQ protein complex exhibited the first principal component separated as two clusters, namely the positive fluctuations and negative fluctuations spread as a dense scatter plot ranging from –300 to 100, indicating more flexibility in the domains with an average PC 1 trace value at 50 and –190, respectively. The Apo protein also showed a similar trend but to a lesser extent, with an average PC1 trace value of 25 and –120, respectively. However, in the case of Kumudine B, the principal motions were slightly less compared to Apo protein, with an average PC1 trace value of 10 and –70, respectively. Lastly, Picrasamide A, with an average PC1 trace value of 20 and –10, respectively, and Quassidine I, with an average PC1 trace value of 20 and –20, respectively, showed the very least spread principal motions, suggesting restricted motion of the domains compared to Streptomycin, thus supporting our findings in the previous sections (Fig 10).

### SAR analysis

**Fig 11:**
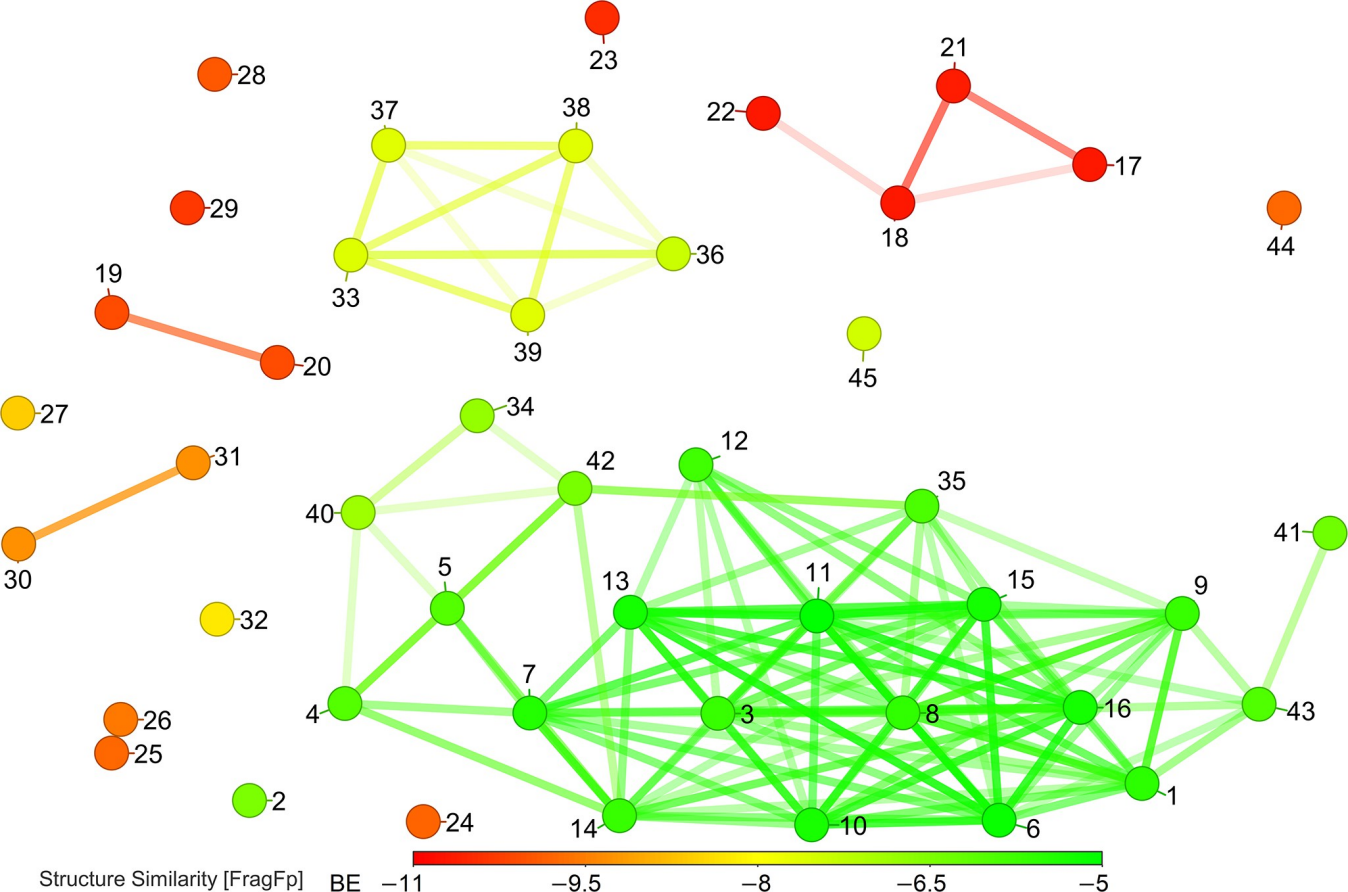
Structural similarity of photochemicals of *P*. *quassioides* and standard drug used in the study. The structurally similar phytochemicals are represented by the lines between the dots and the compound names, as listed in Table 2, are represented by the numbers on each dot.

The SAR analysis is a theoretical model that can calculate the qualitative association between the chemical substructures to exhibit certain biological activities [51]. Extensive detection of the compounds’ SAR would help improve their medicinal properties and minimize their toxicity based on their multiple behaviors (such as stereoisomerism, structural isomerism, etc.) [52]. The molecules with similar structural properties will bind to the same biological targets and occupy the same protein receptor binding sites, assisting in formulating strategies based on shape similarity for their possible use during drug discovery [53]. This structural similarity between the molecules may indicate the same pharmacological effects at the protein receptor. The structural similarity chart was prepared to show the relationship between the canonical SMILES structure and the binding behaviour of phytochemicals used in this study. The chart showed that the phytochemicals were found to be arranged based on their range above 80% structural similarity relationship and same binding affinity range (Fig 11). The structurally analogous phytochemicals of *P*. *quassioides* exhibited comparable binding affinities towards the targeted XopQ protein, occupying analogous 3D positions within the protein binding sites, and demonstrating comparable pharmacological effects at the targeted protein receptor [23]. Further, the structural modifications of the ligand can be implemented to stabilize a molecule in a conformation that is more favorably accommodated by one target over another. This strategy has been demonstrated as effective in enhancing binding affinity [54].

### ADMET properties

The ADMET studies are performed through *in silico* models, which pose no potential harm to animals or humans. The research is conducted via computers, reducing drug discovery costs and improving efficiency in the early stages of drug development [22, 40]. Physicochemical descriptors of the potential compounds of *P*. *quassioides* and standard drug (Streptomycin) were analyzed to evaluate their drug-likeness nature based on the fundamental rule of drug-likeness. The predicted results of physicochemical descriptors are illustrated in Table 4. Interestingly, Quassidine I, Kumudine B and Picrasamide A bear a molecular weight of <500 g/mol with fewer LogP values than Streptomycin. When compared to large molecules, the low molecular weight (<500 g/mol) molecules are more easily transported, dispersed and absorbed by the cell membrane [55]. The positive LogP values also specify that the compounds can more easily pass through the cell membrane and interact with the biomolecules to inhibit metabolic enzyme function [56]. Therefore, it is essential to calculate the LogP values of the selected compounds with its acceptable limit of <5, which is predicted as ideal lipophilicity for crossing the pathogen cell membrane. According to Lipinski’s rule of Five, most drugs have ≤10 hydrogen bond acceptors and ≤5 donors. Quassidine I, Kumudine B, and Picrasamide A qualify under these rules compared to Streptomycin. Moreover, the TPSA correlates with passive drug transport via membranes and enables the predictions of Caco-2 permeability, human intestinal absorption, and blood-brain barrier (BBB) penetration of drugs [57]. The compounds with TPSA values <100 Å^2^ have good absorption and membrane permeability. The compounds with TPSA values >140 Å^2^ have a strong polarity and cannot absorb. The observed TPSA of Quassidine I, Kumudine B and Picrasamide A were less than Streptomycin, which indicates that the body may absorb them. Quassidine I, Kumudine B, and Picrasamide A were found to qualify for most of the drug-likeness rules compared to Streptomycin, which makes it a promising drug-like candidate.

**Table 4:** Physicochemical descriptors of lead phytochemicals of *P*. *quassioides* and standard drug.

Descriptor	Quassidine I	Kumudine B	Picrasamide A	Streptomycin
Molecular weight (g/mol)	431.563	494.507	398.415	581.58
LogP	6.29336	4.9305	2.0129	–8.1611
Rotatable bonds	1	7	8	9
Hydrogen bond acceptors	1	7	6	15
Hydrogen bond donors	2	3	2	12
TPSA (Å^2^)	48.35	130.19	95.12	336.43

Moreover, predicting the physicochemical descriptors of potential compounds influences their ADMET properties of compounds, which are necessary to develop novel drugs with desired biological activities. The results obtained from the online pkCSM pharmacokinetics property prediction are shown in Table 5. The absorption properties studied were water solubility, colon carcinoma cell line (Caco-2) permeability, human intestinal absorption, P-glycoprotein interactions and skin permeability. The LogS measures the number of drugs that can dissolve in water at 25 °C and is directly related to the drug’s water solubility [58]. The water solubility of Kumudine B was almost the same as Streptomycin.

**Table 5:** ADMET properties of lead phytochemicals of *P*. *quassioides* and standard drug.

	Model Name	Quassidine I	Kumudine B	Picrasamide A	Streptomycin
**Absorption**	Water solubility (log mol/L)	– 3.518	–2.984	– 3.703	–2.891
	Caco-2 permeability (log P_app_ in 10^−6^ cm/s)	0.754	1.765	1.08	0.08
	Human intestinal absorption (% Absorbed)	93.17	84.472	83.504	0
	Skin permeability (log Kp in cm/h)	– 2.736	–2.735	– 2.872	–2.735
	P-glycoprotein substrate	Yes	No	Yes	Yes
	P-glycoprotein I inhibitor	Yes	Yes	Yes	No
	P-glycoprotein II inhibitor	Yes	Yes	No	No
**Distribution**	Human VDss (log L/kg)	0.496	–0.435	0.018	–0.351
	Human fraction unbound (Fu)	0.176	0.17	0.064	0.543
	BBB permeability (log BB)	0.508	–1.629	– 0.641	–2.031
	CNS permeability (log PS)	– 0.485	–3.902	– 3.327	–6.492
**Metabolism**	CYP2D6 substrate	Yes	No	No	No
	CYP3A4 substrate	Yes	No	Yes	No
	CYP1A2 inhibitor	Yes	No	No	No
	CYP2C19 inhibitor	No	No	Yes	No
	CYP2C9 inhibitor	No	Yes	No	No
	CYP2D6 inhibitor	Yes	No	No	No
	CYP3A4 inhibitor	Yes	Yes	Yes	No
**Excretion**	Total clearance (log mL/min/kg)	1.423	0.56	0.324	0.005
	Renal OCT2 substrate	No	No	No	No
**Toxicity**	AMES toxicity	Yes	No	Yes	Yes
	Human Max. tolerated dose (log mg/kg/day)	0.453	0.544	– 0.218	0.569
	hERG I inhibitor	No	No	No	No
	hERG II inhibitor	Yes	Yes	Yes	Yes
	Oral Rat Acute Toxicity (LD_50_) (mol/kg)	2.259	2.461	2.06	2.471
	Oral Rat Chronic Toxicity (LOAEL) (log mg/kg bw/day)	1.501	1.981	1.356	4.229
	Hepatotoxicity	Yes	Yes	Yes	No
	Skin sensitisation	No	No	No	No
	*T*. *pyriformis* toxicity (log μg/L)	0.285	0.285	0.41	0.285
	Minnow toxicity (log mM)	– 0.51	–2.42	0.301	11.256

Permeability coefficients across the Caco-2 monolayers are generally used to calculate the intestinal permeability and absorption of drugs in humans [59]. The obtained permeability coefficient (Papp) value is > 8 × 10^−6^ cm/s for the compounds believed to have high Caco-2 permeability and is completely absorbed from the human intestine. Quassidine I, Kumudine B, and Picrasamide A were considered to have more Caco-2 permeability as they have a greater Papp value than Streptomycin. Human intestinal absorption significantly affects drug bioavailability while developing new drug substances [60]. The classification of compounds with low or high fractional absorption of ≤ 30% or > 30%, respectively, is based on the intestinal absorption percentage prediction. Quassidine I, Kumudine B and Picrasamide A were predicted to show good human intestinal absorption compared to Streptomycin. Skin permeability is the rate at which chemical substances penetrate across the outermost layer of the epidermis (or stratum corneum) [61]. The significance of skin absorption is denoted by the human skin permeation coefficient (log Kp) value, which is extensively used to quantify the transportation of small molecules in the stratum corneum. Quassidine I, Kumudine B, Picrasamide A and Streptomycin showed relatively high human skin permeability as they had a log Kp value <–2.5 cm/h. The human ATP-binding cassette (ABC) transporter P-glycoprotein is crucial for the absorption and excretion of drugs [62]. The P-glycoprotein substrates are transported from the cell by P-glycoprotein, while the P-glycoprotein inhibitors slow down the P-glycoprotein transport activity. Kumudine B was not a substrate of P-glycoprotein and couldn’t be expelled from the cells by P-glycoprotein. However, Quassidine I, Kumudine B and Picrasamide A were predicted to be P-glycoprotein inhibitors compared to Streptomycin. Quassidine I, Kumudine B and Picrasamide A were promising drugs for enhancing oral absorption and bioavailability due to their P-glycoprotein inhibitory effect.

The distribution of drugs is the second stage of pharmacokinetics. It is imperative to disperse the drug to reach its intended target site of action in the human body by passing through the blood flow [63]. The drug distribution properties included the volume of distribution (VDss), fraction unbound (Fu), blood-brain barrier (BBB) permeability and central nervous system (CNS) permeability. Apparent VDss is the theoretical volume available for the drug to equally disperse in the body with the blood flow [64]. The predicted VDss of Kumudine B was relatively low as it has a log VDss value of <–0.15 L/kg. However, Kumudine B has been distributed more than Streptomycin. The unbound (free) drug concentration in plasma is an important measure used to evaluate the therapeutic efficacy of drugs at the therapeutic target site of a pharmacological effect in pharmacokinetic and pharmacodynamic analysis [65]. Quassidine I, Kumudine B and Picrasamide A, with the Fu values between 0.05 and 0.2 were considered to have a moderate unbound fraction in plasma compared to Streptomycin. The prediction of BBB permeability of drugs is a key factor in drug development to avoid brain uptake through the selective semi-permeable membrane barrier between the blood and brain [66]. Kumudine B, which showed a log BB value of <–1, was difficult to cross the BBB to target other body parts and prevent the possible psychotropic side effects in CNS. Log PS is a brain pharmacokinetic value that directly measures the ability of drug penetration into the CNS [67]. The Kumudine B and Picrasamide A with log PS value of <–3 might not enter the brain.

Most drugs and other lipophilic xenobiotics can undergo oxidative biotransformation to facilitate their excretion with the help of the body’s cytochromes P450 (CYPs) enzymes [68]. The metabolism properties were inhibitor and cytochrome P450 (CYP450) enzyme substrate. Compared to Streptomycin, Quassidine I and Picrasamide A were most likely metabolized by CYP450 in the liver, but Quassidine I, Kumudine B and Picrasamide A were predicted to be CYP450 inhibitors. Drug excretion is the process used to remove drugs from the body either in their unchanged form or as active/inactive biotransformed metabolites [69]. The excretion properties were the total clearance from the liver and bile duct and renal organic cation transporter 2 (OCT2) clearance from the kidney. The results confirmed that the predicted total clearance of Quassidine I, Kumudine B and Picrasamide A was more than Streptomycin and not the OCT2 substrate.

In addition, the predicted toxicity parameters of the drug were AMES toxicity, maximum human tolerated dose, human Ether-à-go-go Related Gene (hERG) I and II inhibitors, oral rat acute toxicity (LD_50_) and chronic toxicity (LOAEL), hepatotoxicity, skin sensitization, *Tetrahymena pyriformis* toxicity and minnow toxicity. Ames test is a robust bacterial bioassay used to identify possible carcinogens by studying their mutagenic effect on bacteria [70, 71]. The predicted results suggested that Kumudine B was expected to be AMES test negative; thus, it was non-mutagenic and may not act as a carcinogen compared to Streptomycin. The maximum tolerated dose of Kumudine B was considered high, with 0.544 log mg/kg/day causing minimal side effects in humans. Besides, Quassidine I, Kumudine B, Picrasamide A and Streptomycin could inhibit the hERG II channel but not the hERG I channel. Kumudine B and Streptomycin, with higher lethal dose (LD_50_) values, were less hazardous and posed lower toxicity to humans and animals. The minimal toxic concentration (LOAEL) of Quassidine I, Kumudine B, and Picrasamide A was less than that of Streptomycin. Quassidine I, Kumudine B and Picrasamide A may be hepatotoxic but not have the skin-sensitizing potential. However, Quassidine I and Kumudine B showed the same *T*. *pyriformis* toxicity value and lowest minnow toxicity compared to Streptomycin.

**Fig 12:**
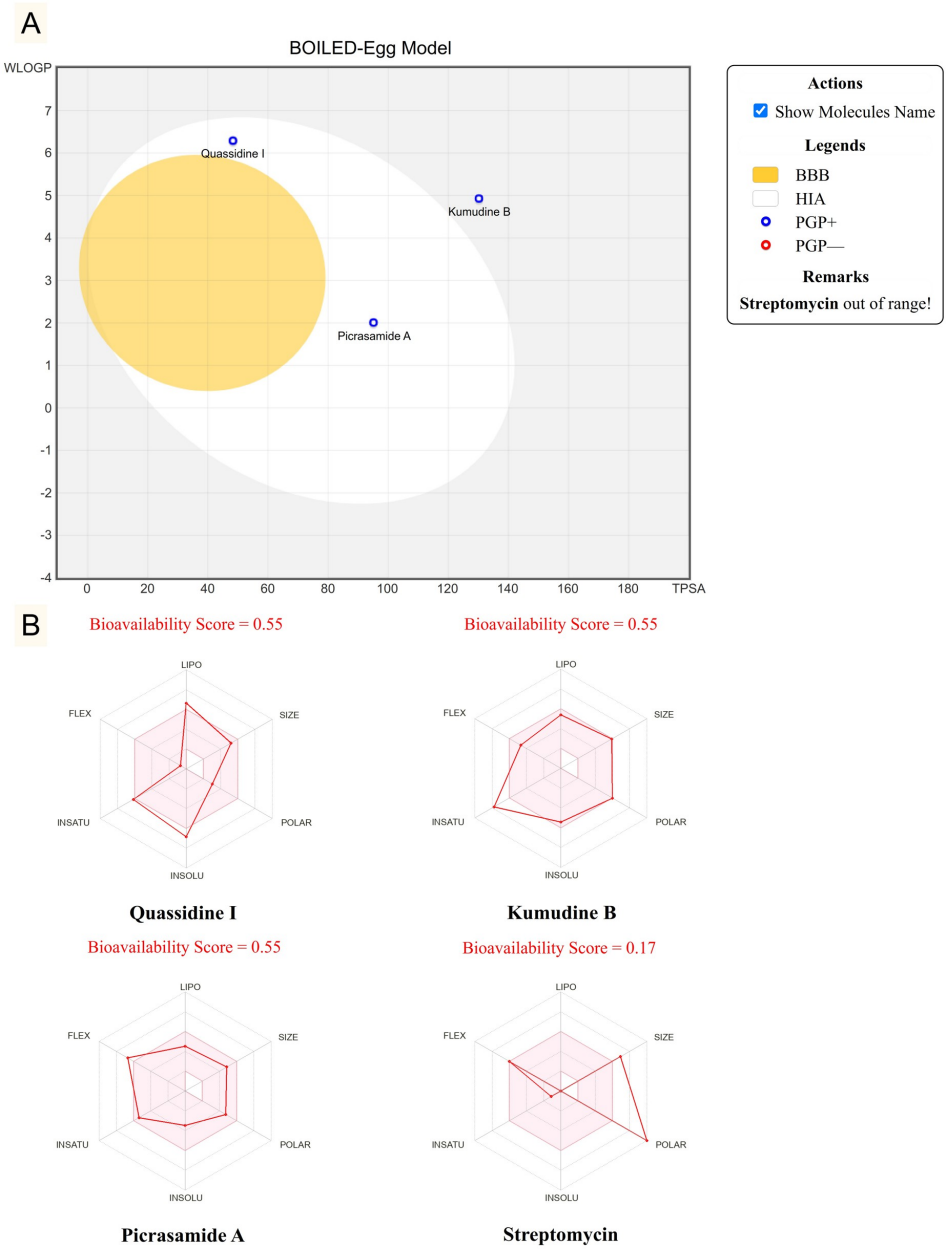
BOILED-Egg predictive model (A) and Bioavailability score (B) of lead phytochemicals of *P*. *quassioides* and standard drug. White region: a high probability of human intestinal absorption (HIA) and yolk (yellow) region: a high probability of brain penetration (BBB). Blue dots are P-glycoprotein substrates (PGP+) and red dots are P-glycoprotein non-substrate (PGP−).

BOILED-Egg model is the most efficient predictive tool used in drug discovery to calculate the passive gastrointestinal absorption and brain penetration (BBB) of chemical substances based on the function of their position in the graph plotted with lipophilicity (WLOGP) versus polarity (TPSA) [36]. The white and yolk (yellow) regions are the physicochemical spaces where the chemical substances have the highest possibility of gastrointestinal absorption and brain penetration, respectively. In addition, the chemical substances predicted to be actively effluxed from the CNS by P-glycoprotein (PGP+) and non-substrate of P-glycoprotein (PGP−) are indicated by the blue and red points, respectively. The BOILED-Egg model is illustrated in Fig 12A. Quassidine I and Picrasamide A had the highest possibility of gastrointestinal absorption. Kumudine B could not be absorbed through the gastrointestinal tract and BBB was non-permeant as it was outside the Egg and in the grey region. However, Streptomycin did not have gastrointestinal absorption, and BBB was non-permeant because it was further outside the plot range. Quassidine I, Kumudine B and Picrasamide A were actively effluxed from the CNS by P-glycoprotein (PGP+).

Drug bioavailability is another primary pharmacokinetic parameter that quantifies the proportion of the drug absorbed and available in a pharmacologically active form at the intended target site of action to cause systemic effects [72]. The biological activity of the compounds can be measured in terms of their bioavailability score as the targeted protein inhibitors. The drugs were classified into three different groups: the drug molecules having a bioavailability score greater than 0 were found to show more biological activity; the drug scores extending from −0.5 to 0 were expected to be considered moderately active; and the drugs were presumed to be inactive if the score was less than −0.5. Quassidine I, Kumudine B and Picrasamide A had a bioavailability score of 0.55 for enzyme inhibitors, thus considered biologically more active than Streptomycin (Fig 12B). Therefore, the findings suggested that Quassidine I, Kumudine B and Picrasamide A extracted from *P*. *quassioides* merit further *in vitro* and *in vivo* evaluation to consider them as potential antibacterial agents to control the *X*. *oryzae* infection.

## Conclusion

Managing bacterial blight disease caused by *X*. *oryzae* with natural compounds is a cutting-edge and risk-free application. By assessing more natural chemicals and their interactions with *X*. *oryzae*, the researchers might uncover novel antibacterial drugs that demonstrate increased efficiency and decrease the toxicity and risk of resistance development. The present study revealed that Kumudine B, Picrasamide A, Quassidine I and Quassidine J isolated from *P*. *quassioides* showed the most significant binding affinity towards the closed state of XopQ protein than the other compounds tested and reference standard drug (Streptomycin) by chemoinformatic analysis. The MD analysis confirmed the stability of top lead ligands (Kumudine B, Picrasamide A, and Quassidine I)-bound XopQ protein complex with slightly lower fluctuation than Streptomycin. The MM-PBSA calculation also confirmed the strong interactions of top lead ligands (Kumudine B and Quassidine I) with XopQ protein, as they possessed the least binding energy. The ADMET analysis showed that Quassidine I, Kumudine B, and Picrasamide A were found to qualify for most of the drug-likeness rules with excellent bioavailability scores compared to Streptomycin. The current study has provided useful insights and target compounds against *X*. *oryzae* infection through *in silico* evaluation and validation. However, future studies should focus on purification and experimental testing of the lead compounds (Kumudine B, Picrasamide A, and Quassidine I) by additional *in vitro* and *in vivo* experimentation to establish their effectiveness and safety against *X*. *oryzae* infection.

## Supporting information

S1 Table Canonical SMILES strings of phytochemicals from *P*. *quassioides* and standard drug. (PDF)

S1 Fig Snapshot of (A) Picrasamide A and (B) Quassidine I at 0 ns, 100 ns, 200 ns and 300 ns, respectively. (JPG)
