# Supplementary material for: Exploration of Type III effector Xanthomonas outer protein Q (XopQ) inhibitor from Picrasma quassioides as an antibacterial agent using chemoinformatics analysis
Source: PLoS One. 2024 Jun 18;19(6):e0302105. doi: 10.1371/journal.pone.0302105 (PMC11185476; doi:10.1371/journal.pone.0302105)
Supplement: S1 Table — (PDF) [file pone.0302105.s001.pdf]

Supplementary data

Supplementary Table 1. Canonical SMILES strings of phytochemicals from *P. quassioides* and standard drug

Sl. No.	Compound Name	PubChem CID	Canonical SMILES string
1	Bruceantin	CID_5281304	CC1=C(C(=O)CC2(C1CC3C45C2C(C(C(C4C(C(=O)O3)OC(=O)C=C(C)C(C)C)(OC5)C(=O)OC)O)O)C)O
2	* Kumulactone A	-	CC1C=C(C)C(=O)C2(C)C1CC3OC(=O)CC4(C)C(C)C(C)C(OC(=O)C5=CC6=C(OCO6)C(=C5)C)C2C34C
3	* Kumulactone B	-	CC1C=C(C)C(=O)C2(C)C1CC3OC(=O)CC4(O)C(C)C(C)C(O)C2C34C
4	* Picrasinoside J	-	CC1C=C(C)C(=O)C2(C)C1CC3OC(CC4C(C)(O)C(=O)C(O)C2C34C)OC5OC(CO)C(O)C(O)C5O
5	* Picrasinoside K	-	CC1C=C(C)C(=O)C2(C)C1CC3OC(CC4C(C)(O)C(=O)C(O)C2C34C)OC5OC(CO)C(O)C(O)C5O
6	Quassine	CID_65571	CC1C=C(C(=O)C2(C1CC3C4(C2C(=O)C(=C(C4CC(=O)O3)C)OC)C)C)OC
7	Neoquassine	CID_72964	CC1C=C(C(=O)C2(C1CC3C4(C2C(=O)C(=C(C4CC(O3)O)C)OC)C)C)OC
8	Simalikalactone D	CID_6711208	CCC(C)C(=O)OC1C2C3(C(C(C4C2(CO3)C(CC5C4(C(C(=O)C=C5C)O)C)OC1=O)O)O)C
9	Simalikalactone E	CID_53324651	CCC(C)C(=O)OC1C2C(=CC(=O)C(C2(C3C(C(C4(C5C3(C1OC(=O)C5OC(=O)C(C)CC)CO4)C)O)O)C)O)C
10	Picrasin A	CID_185611	CC1C=C(C(=O)C2(C1CC3C4(C2C(CC(C4CC(=O)O3)(C)C(=O)C5CC(=O)OC5)O)C)C)OC
11	Picrasin B	CID_12313355	CC1CC(C(=O)C2(C1CC3C4(C2C(=O)C(=C(C4CC(=O)O3)C)OC)C)C)O
12	Picrasin C	CID_182145	CC1CC(C(=O)C2(C1CC3C4(C2C(C(C(C4CC(=O)O3)C)OC)OC(=O)C)C)C)O
13	* 12-hydroxyquassin	-	CC1C=C(C)C(=O)C2(C)C1CC3OC(=O)CC4C(=C(O)C(=O)C2C34C)C
14	Nigakihemiacetal A	CID_441803	CC1C=C(C(=O)C2(C1CC3C4(C2C(C(C(C4CC(O3)O)(C)O)OC)O)C)C)OC
15	Nigakilactone A	CID_10452259	CC1C=C(C(=O)C2(C1CC3C4(C2C(C(C(C4CC(=O)O3)C)O)O)C)C)OC
16	Nigakilactone B	CID_12313347	CC1C=C(C(=O)C2(C1CC3C4(C2C(C(C(C4CC(=O)O3)C)OC)O)C)C)OC
17	* 1-hydroxymethyl-8-hydroxy-beta-carboline	-	CC1=C2NC3=C(C=CC=C3O)C2=CC=N1
18	Dehydrocrenatidine	CID_5318875	COC1=CC=CC2=C1NC3=C2C(=CN=C3C=C)OC
19	* Quassidine I	-	CC1=C2[NH]C3=C(C(=C[N+]4=C3CCC(C4)C5=NC=C(C)C6=C5[NH]C7=CC=CC=C67)C)C2=CC=C1
20	* Quassidine J	-	CC1=C2[NH]C3=C(C(=C[N+]4=C3CCC(C4)C5=NC=C(C)C6=C5[NH]C7=CC=CC=C67)C)C2=CC=C1
21	Picrasidine I	CID_5324360	COC1=CN=C(C2=C1C3=C(N2)C(=CC=C3)O)C=C
22	* 6,12-dimethoxy-3-ethyl-beta-carboline	-	CCC1=C2NC3=C(C)C=CC=C3C2=C(C)C=N1

23	* Kumudine A	-	COC(\C=C\C1=CC(=C(O)C(=C1)OC)OC)C2=NC=CC3=C2[NH]C4=CC=CC=C34
24	* Kumudine B	-	COC(CC(=O)C1=C2[NH]C3=C(C=CC=C3)C2=CC=N1)C(=O)C4=NC=C(OC)C5=C4[NH]C6=C(O)C=CC=C56
25	Nigakinone	CID_5320161	COC1=C(C(=O)N2C3=CC=CC=C3C4=C2C1=NC=C4)O
26	Methyl nigakinone	CID_638215	COC1=C(C(=O)N2C3=CC=CC=C3C4=C2C1=NC=C4)OC
27	Picrasidine O	CID_5320558	CN1C=CC2=C3C1=C(C(=O)C(=O)N3C4=CC=CC=C24)OC
28	* 4,5-dimethoxy-10-hydroxycanthin-6-one	-	CC1=C(C)C2=NC=CC3=C2N(C2=CC=C(O)C=C32)C1=O
29	* 8-hydroxycanthin-6-one	-	OC1=C2N3C4=C(C=CN=C4C=CC3=O)C2=CC=C1
30	* Picrasmalignan A	-	CC1=C(O)C=CC(=C1)C2OC3=C(C=C(C=C3C)C4OC5=C(C=C(\C=C\C=O)C=C5C)C4CO)C2CO
31	Dehydrodiconiferyl alcohol	CID_5372367	COC1=CC(=CC2=C1OC(C2CO)C3=CC(=C(C=C3)O)OC)C=CCO
32	Picraquassioside C	CID_10077272	COC1=CC(=CC(=C1OC(CO)C(C2=CC(=C(C(=C2)OC)OC3C(C(C(C(O3)CO)O)O)O)OC)O)OC)C=CCO
33	* Picraquassin A	-	CC(C)(O)C1C(O)CC(C1O)C2CC=C3C2(C)CCC4C5(C)CCC(O)C(C)(C)C5CC(O)C34C
34	* Picraquassin B	-	CCOC1OC(CC1C2CC=C3C2(C)CCC4C5(C)CCC(O)C(C)(C)C5CC(O)C34C)C=C(C)C
35	* Picraquassin C	-	CCCCCC(=O)OC1CC2(C)C(CCC2C3(C)C(O)CC4C(C)(C)C(=O)C=CC4(C)C13)C5(O)CC(OC5OC)C(O)C(C)(C)O
36	* Picraquassin D	-	CC(CC(O)C(O)C(C)(C)O)C1CC=C2C1(C)CCC3C4(C)CCC(=O)C(C)(C)C4CC(O)C23C
37	* Picraquassin E	-	CC1CC(CC1C2CCC3(C)C4=CCC5C(C)(C)C(O)CCC5(C)C4CCC23C)C=C(C)C
38	* Picraquassin I	-	CC1CC(CC1C2CCC3(C)C4=CCC5C(C)(C)C(O)CCC5(C)C4CCC23C)C=C(C)C
39	* Picraquasssin J	-	CC1CC(CC1C2CCC3(C)C4=CCC5C(C)(C)C(O)CCC5(C)C4CCC23C)C=C(C)C
40	* Picraquassin K	-	CC1OC(CC1C2CCC3(C)C4=CCC5C(C)(C)C(O)CCC5(C)C4CCC23C)C(O)C(C)(C)O
41	* Kumuquassin A	-	CC(C)(O)C(O)C1C\C(C(=O)O1)=C2/CCC3(C)C4=CCC5C(C)(C)C(=O)CCC5(C)C4CCC23C
42	* Kumuquassin B	-	CC(C)(O)C(O)C1OC(O)C2(OC12)C3CCC4(C)C5=CCC6C(C)(C)C(=O)CCC6(C)C5CCC34C
43	* Kumuquassin C	-	CC(C)(O)C(O)C1OC(=O)C(=C1)C2CCC3(C)C4=CCC5C(C)(C)C(=O)CCC5(C)C4CCC23C
44	* Picrasamide A	-	O=C(CCC1=CC2=C(OCO2)C=C1)NCCCNC(=O)C1=CC2=C(OCO2)C=C1
45	Streptomycin [Standard drug]	CID_19649	CC1C(C(C(O1)OC2C(C(C(C(C2O)O)N=C(N)N)O)N=C(N)N)OC3C(C(C(C(O3)CO)O)O)NC)(C=O)O
