# Supplementary figures and images for: Exploration of Type III effector Xanthomonas outer protein Q (XopQ) inhibitor from Picrasma quassioides as an antibacterial agent using chemoinformatics analysis
Source: PLoS One. 2024 Jun 18;19(6):e0302105. doi: 10.1371/journal.pone.0302105 (PMC11185476; doi:10.1371/journal.pone.0302105)

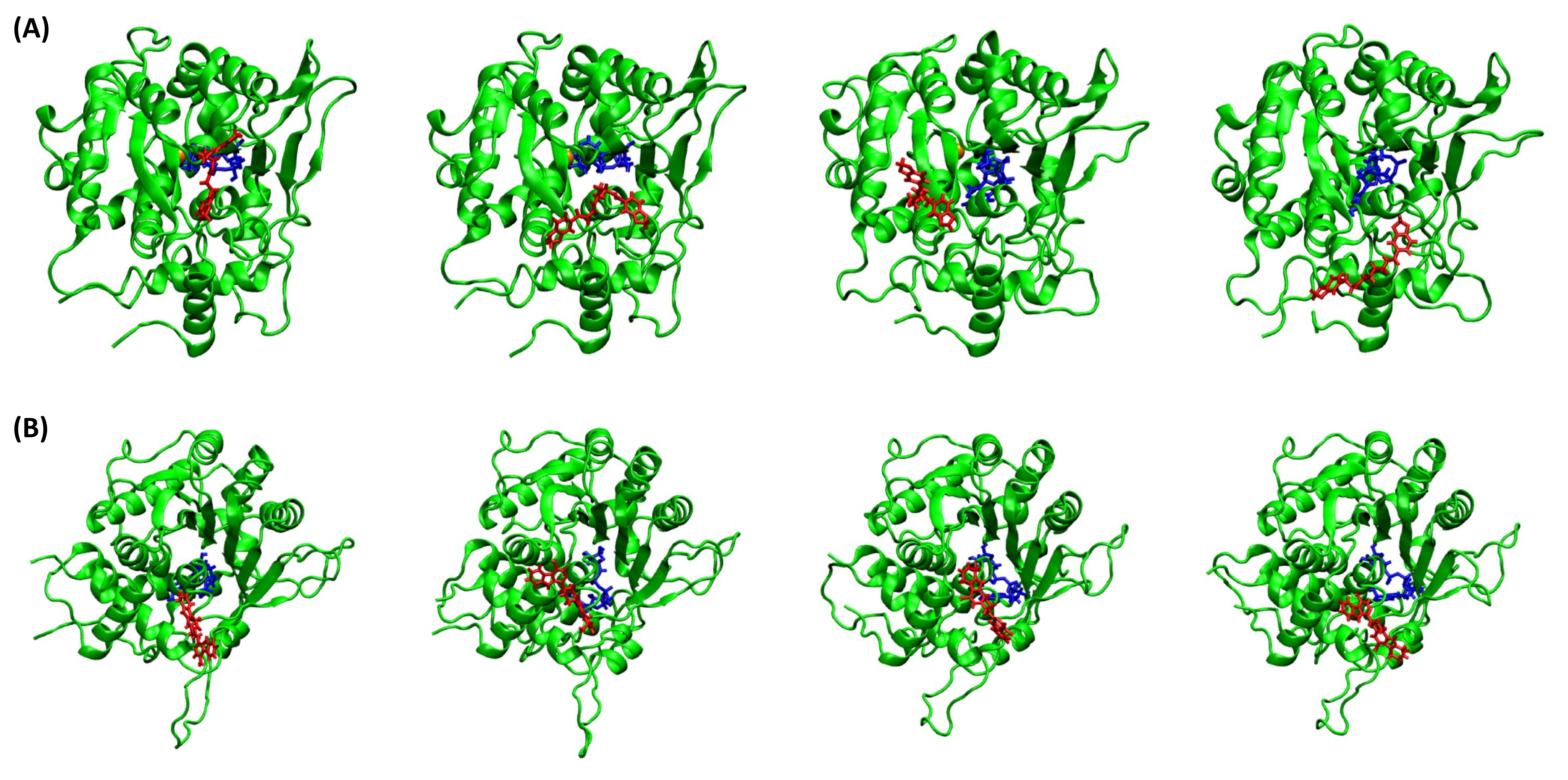

Supplement: S1 Fig — (JPG) [file pone.0302105.s002.jpg]
